# Development of a hemolysis filter system for the selective removal of free hemoglobin, haem and iron from blood envisaged for use in extracorporeal circuits

**DOI:** 10.1038/s41598-026-57088-y

**Published:** 2026-07-01

**Authors:** Noora Naghavi, Lisa Barbaro, Mark JD. Griffiths, Simon Davidson, Daniel D. Melley, John O’Neil, Karl Riley, Richard W. Issitt, Gregory J. Quinlan, Nathan A. Davies, Alan C. Spivey

**Affiliations:** 1 National Heart and Lung Institute (NHLI), Imperial College London, Guy Scadding Building, 70 Cale Street, London, SW3 6PT UK; 2 Department of Chemistry, Molecular Science Research Hub (MSRH), Imperial College London, White City Campus, 82 Wood Lane, London, W12 0BZ UK; 3 Warren Centre for Neuroscience Drug Discovery, Vanderbilt University, Nashville, TN 37232 USA; 4 Department of Pharmacology, Vanderbilt University School of Medicine, Nashville, TN 37232 USA; 5 St Bartholomew’s Hospital, West Smithfield, London, EC1A 7BE UK; 6 Faculty of Medical Science, Rayne Institute, University College London, 5 University Street, London, WC1E 6JF UK; 7 Institute for Liver and Digestive Health, UCL Meical School, Royal Free Campus, Roland Hill Street, London, NW3 2PF UK; 8 Department of Children’s Cardiovascular Disease, UCL Institute of Cardiovascular Science, Zayed Centre for Research into Rare Disease in Children, 20 Guilford Street, London, WC1N 1DZ UK

**Keywords:** Hemolysis, Extracorporeal blood purification, Affinity filtration, Hemocompatibility biomaterials, Cell-free hemoglobin removal, Cardiopulmonary bypass, Biochemistry, Biological techniques, Biotechnology, Medical research

## Abstract

Hemolysis, the rupture of red blood cells, releases hemoglobin, haem, and redox-active iron into the bloodstream. When the body’s scavenging capacity is overwhelmed, these species can exert deleterious effects, as seen in hemolytic disorders. Hemolysis can also occur in extracorporeal circuits due to mechanical forces acting on blood during circulation. To mitigate these effects, we developed a filter designed for integration into extracorporeal circuits that is designed to capture hemolysis-associated byproducts using immobilised ligands. The filter prototype comprises glyoxal-functionalized agarose beads covalently immobilized with three binding agents: haptoglobin (Hp, binds cell-free hemoglobin [cfHb]), human serum albumin (HSA, binds haem), and desferrioxamine (DFO, binds free iron). Ligand immobilization was optimized to achieve strong covalent attachment to the agarose matrix. Optimized immobilization produced high ligand loading per milliliter of beads (mean ± SD): Hp 74.4 ± 11.7 mg/mL, HSA 84.6 ± 8.2 mg/mL, and DFO 43.3 ± 4.8 mg/mL. In vitro studies showed removal capacities of 12.7 ± 0.2 mg/mL for cfHb, 2708.5 ± 18.5 µg/mL for haem, and 309.7 ± 13.7 µg/mL for iron. The system retained binding activity in plasma and hemolyzed whole blood, and no increase in TAT or D-dimer was detected under the tested ex vivo conditions. These findings demonstrate the potential of this affinity-based filtration system to reduce hemolysis-associated complications in extracorporeal circulation.

**Supplementary Information:**

The online version contains supplementary material available at https://doi.org/10.1038/s41598-026-57088-y.

## Introduction

Iron is a valuable but limited biological resource. Dietary iron uptake is tightly regulated and typically insufficient to meet daily physiological demands; thus, iron is recycled primarily rather than excreted^1–3^. However, iron is also a potentially reactive substance capable of catalyzing the formation of damaging reactive oxygen species (ROS), promoting microbial growth, and altering cell fate upon uptake^4,5^.

To limit such potential adverse outcomes, the recycling of iron resources is tightly regulated systemically *via* liver-mediated mechanisms and locally at the cellular level^6,7^. A series of extracellular and intracellular protein-binding agents and receptors facilitate iron recycling. The compartmentalization of iron resources provides further protection against unwanted consequences. The iron/haem transport protein hemoglobin (Hb) is contained within red blood cells (RBCs), which facilitates the transport of oxygen and carbon dioxide but is protected from other biological activities.

During hemolytic episodes, RBCs rupture and release Hb into the plasma where it dissociates into dimers. These dimers can release free haem and ultimately redox-active iron (Fe³⁺)^8–10^. All three iron-containing agents are potentially harmful because they are not compartmentalized within RBCs. Endogenous protective mechanisms bind and retain these agents in safe, nonreactive forms and enable compartmentalization in other cell types. Hb dimers are bound by the plasma protein haptoglobin (Hp), haem by hemopexin and albumin, and iron by transferrin and lactoferrin^11–13^. However, these are finite endogenous resources, and once overwhelmed, adverse consequences can ensue, including vascular and organ injury, clotting disorders, and potential infections^14–16^.

**Fig. 1:**
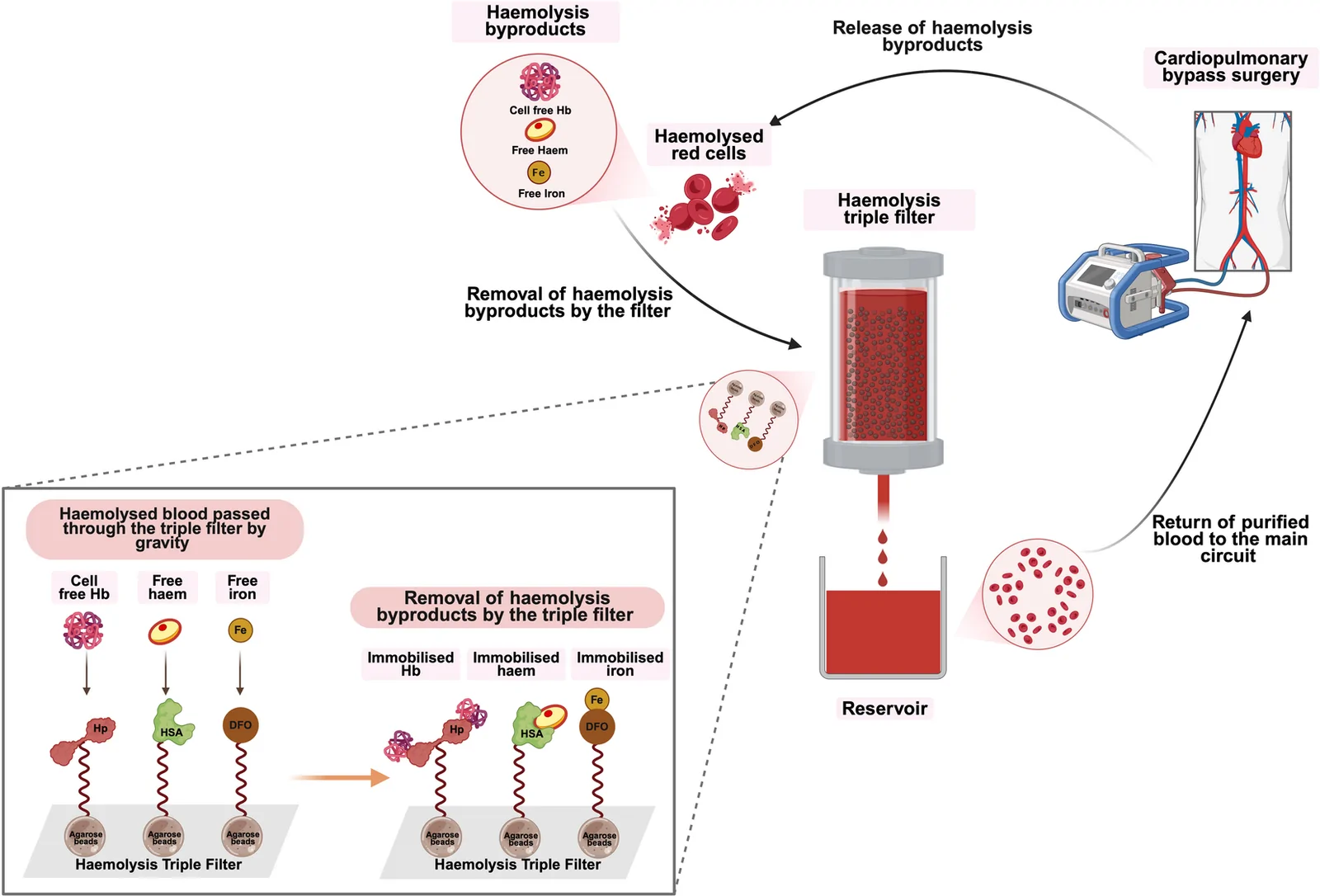
Schematic overview of a hemolysis-targeting triple-affinity filter for use in an extracorporeal circuit. Cardiopulmonary bypass-associated hemolysis releases cfHb, free haem, and labile iron into the circulation. Hemolyzed blood was diverted through a gravity-driven filter packed with glyoxal agarose beads functionalized with haptoglobin (Hp), human serum albumin (HSA), and desferrioxamine (DFO), enabling the capture of cfHb, haem, and iron. The purified blood is then returned to the extracorporeal circuit to reduce the amount of circulating hemolytic byproducts.

Such potential scenarios commonly occur in clinical settings, where extracorporeal circuits are used. These include chronic applications such as kidney dialysis, prolonged support systems such as extracorporeal membrane oxygenation (ECMO), various forms of ventricular assist devices (VADs), and cardiopulmonary bypass (CPB), which are the mainstays of most cardiac surgeries^17–19^. In addition, extracorporeal circuits are employed in the retrieval and reinfusion of shed blood during surgical procedures or in cases of severe trauma. During CPB, blood suctioned from the surgical field is collected through a secondary circuit leading to the cardiotomy reservoir prior to return to the main circuit. Incorporation into this circuit of a purification device in the form of a gravity-fed filter offers the prospect of rermoving the three harmful iron-containing byproducts of hemolysis from the most suction-damaged fraction of the blood and before it is returned to the patient^20–22^. This positioning of the filter device circumvents the need for the filter to operate under the continuous flow conditions present in the main circuit (Fig. 1).

Several strategies have been explored in the literature to mitigate or remove hemolysis-associated byproducts in extracorporeal settings. Conventional approaches use dialysis and hemofiltration membranes, as well as hemoadsorption systems, which can remove circulating solutes depending on membrane properties or sorbent characteristics. However, these methods are generally limited by nonspecific removal, dependence on molecular size or permeability, and low selectivity for hemolysis-derived species^23–25^.

Membrane-based and microfluidic platforms provide alternative approaches for controlled separation and mass transport, including porous micro- and nano-channel systems that exploit size exclusion, porous-wall transport, and electrohydrodynamic or pressure-driven transport phenomena^26–28^. While these systems provide useful models for controlled solute transport, their application to extracorporeal hemolysis management may be limited by throughput constraints, fouling, and lack of molecular specificity for simultaneous cfHb, haem, and iron removal.

Other approaches involve systemic scavenging or chelation strategies, which rely on circulating binding proteins or administered agents to neutralize hemolysis-derived toxins. However, these strategies are not localized to extracorporeal circuits and may be limited by dosing requirements and off-target effects^29,30^.

In contrast, affinity-based approaches offer the potential for selective molecular capture through ligand-specific interactions, enabling targeted removal of hemolysis-derived species such as cfHb, haem, and iron.

We have developed a ‘triple filter’ comprising three specific binding agents: human Hp, which has a strong affinity for cell-free hemoglobin (cfHb; Ka ~ 7 × 10⁵ M⁻¹)^31^; human serum albumin (HSA), which has a significant affinity for haem (Ka ~ 8 × 10⁷ M⁻¹)^32^; and desferrioxamine (DFO), a bacterial siderophore that chelates Fe³⁺ in a 1:1 complex (Ka ~ 1 × 10³⁰ M⁻¹)^33^. To demonstrate the potential of a device with these components, immobilization on aldehyde (glyoxal)-derived agarose beads was performed. Aldehyde-derived agarose beads are stable, biocompatible, and have a broad pore distribution and spherical shape, which allows relatively high flow rates in gravity-fed column processes^34,35^. Each binding agent was covalently immobilized onto agarose beads *via* reductive amination^36^. The resulting functionalized beads were then evaluated experimentally to determine their loading levels (LLs) and target-binding efficiencies. The goal was to mix each functionalized bead type at a ratio within a triple filter column to maximize removal of each target byproduct from hemolyzed blood.

As noted previously, there are multiple applications in relation to the removal of hemolysis products from extracorporeal circuits. The initial design focused on RBC breakdown during cardiac surgery necessitating CPB, especially in relation to blood suctioned from the surgical field, wherein the negative pressure applied directly causes cell lysis^37,38^. The proposed application is not an inline filter in the main pump-driven circuit, but a gravity-fed filtration step in the cardiotomy/suctioned-blood pathway, where blood collected from the surgical field can passes through the filter en route to the cardiotomy reservoir and be subsequently released into the main circuit.

Therefore, we developed and optimized a novel hemolysis triple filter composed of Hp-, HSA-, and DFO-functionalized agarose beads. We report the optimization of the ligand LL and demonstrate the capacity of the beads to sequester their respective hemolytic targets in buffered solutions, human plasma, and hemolyzed whole blood.

## Materials and methods

### Materials

Details of all the materials, reagents, and commercial kits used are provided in the Supplementary Material.

### Immobilization of affinity ligands onto aldehyde-derived agarose beads

**Fig. 2:**
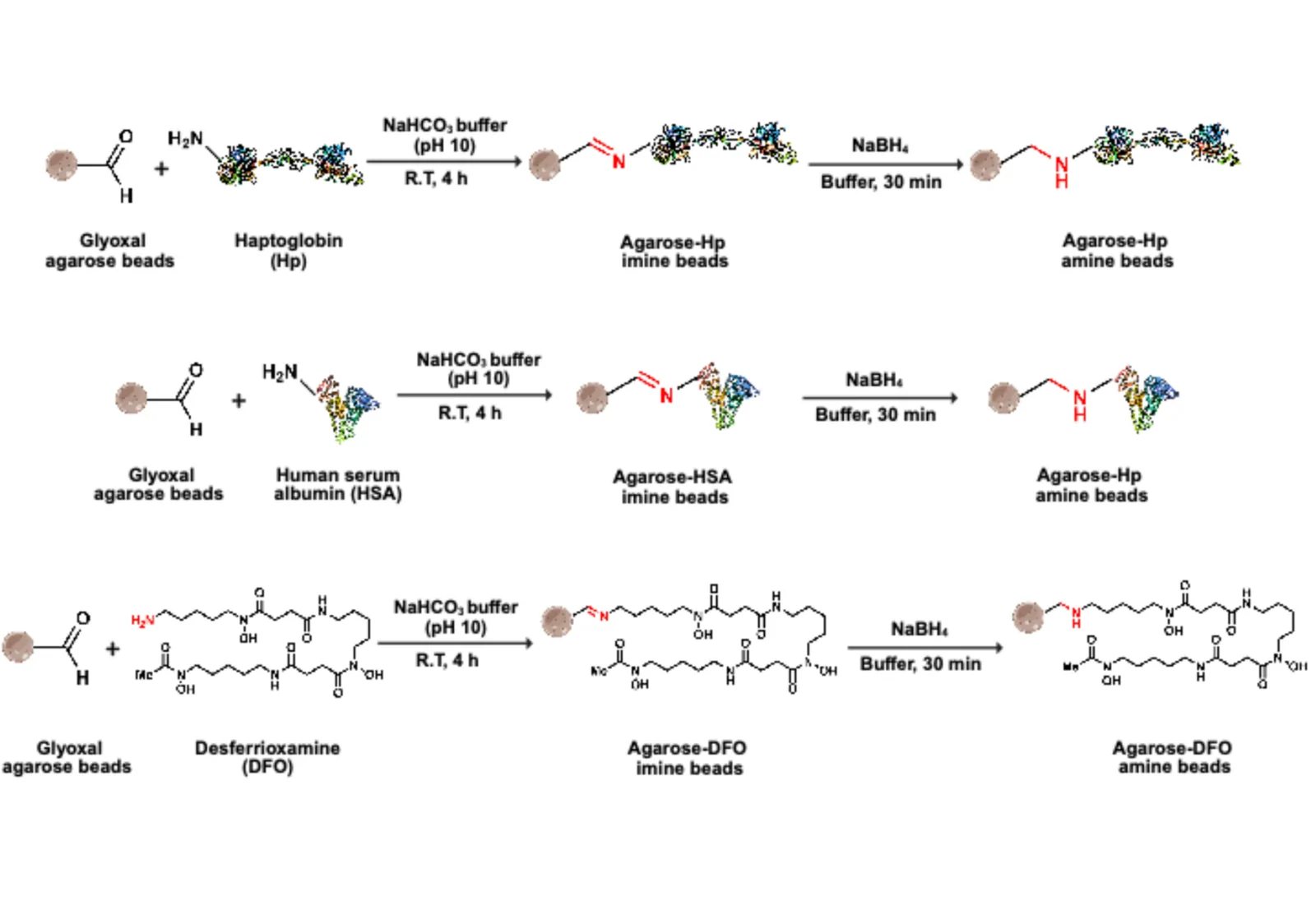
Covalent immobilization of hemolysis-targeting affinity ligands onto glyoxal agarose beads via reductive amination. The primary amine groups on haptoglobin (Hp), human serum albumin (HSA), or desferrioxamine (DFO) react with aldehyde functionalities on glyoxal agarose beads to form Schiff base (imine) linkages, which are subsequently reduced with sodium borohydride (NaBH₄) to yield stable agarose–Hp, agarose–HSA, and agarose–DFO conjugates.

The detailed immobilization protocol is provided in the Supplementary Material. Briefly, Hp, HSA, and DFO were individually immobilized on glyoxal agarose beads *via* reductive amination, forming Schiff base linkages with the aldehyde groups on the agarose matrix, as illustrated in Fig. 2.

### Quantification of affinity ligand loading levels (LLs)

#### Quantification of Hp and HSA LLs

The protein content of the filtrates was analyzed spectrophotometrically via colorimetric Pierce 660 nm and Pierce BCA protein assays on a Clariostar Plus microplate reader (BMG Labtech, Ortenberg, Germany) at 660 and 562 nm, respectively. For calibration, Hp and HSA standard solutions (50–2000 µg/mL) were used to quantify the LL values of Hp and HSA, respectively. The LL was determined as the difference between the initial amount of protein in the stock solution and the amount remaining in the filtrate. All the assays were performed according to the manufacturer’s instructions (*n* = 4 for each case).

#### Quantification of DFO LLs

Fluoraldehyde™ o-phthaldialdehyde (OPA assay): DFO loading was quantified fluorometrically via the Fluoraldehyde™ o-phthaldialdehyde (OPA) reagent (excitation/emission: 340/455 nm) on a Clariostar Plus microplate reader. Standard curves (50–2000 µg/mL DFO) were used, and LL was determined from the difference between pre- and post-coupling DFO concentrations (*n* = 4).

Ferric citrate spectrophotometric assay: DFO immobilization was quantified by converting DFO to iron-saturated ferrioxamine. Briefly, 1 mg/mL (4 mM) ferric citrate was incubated with DFO standards (50–2000 µg/mL), a stock DFO solution (35 mg/mL, 53.2 mM), and post-coupling filtrates for 4 h at RT. The absorbance was measured at 430 nm via a UV‒Vis spectrophotometer (UV‒Vis V-630, JASCO Co. Ltd., Tokyo, Japan). Dilutions were performed to match the concentration of 4 mM ferric citrate. DFO LL was calculated from the calibration curve of ferrioxamine formed from unmodified DFO (*n* = 4).

### Optimization of hemolysis byproduct binding in aqueous buffer

**Fig. 3:**
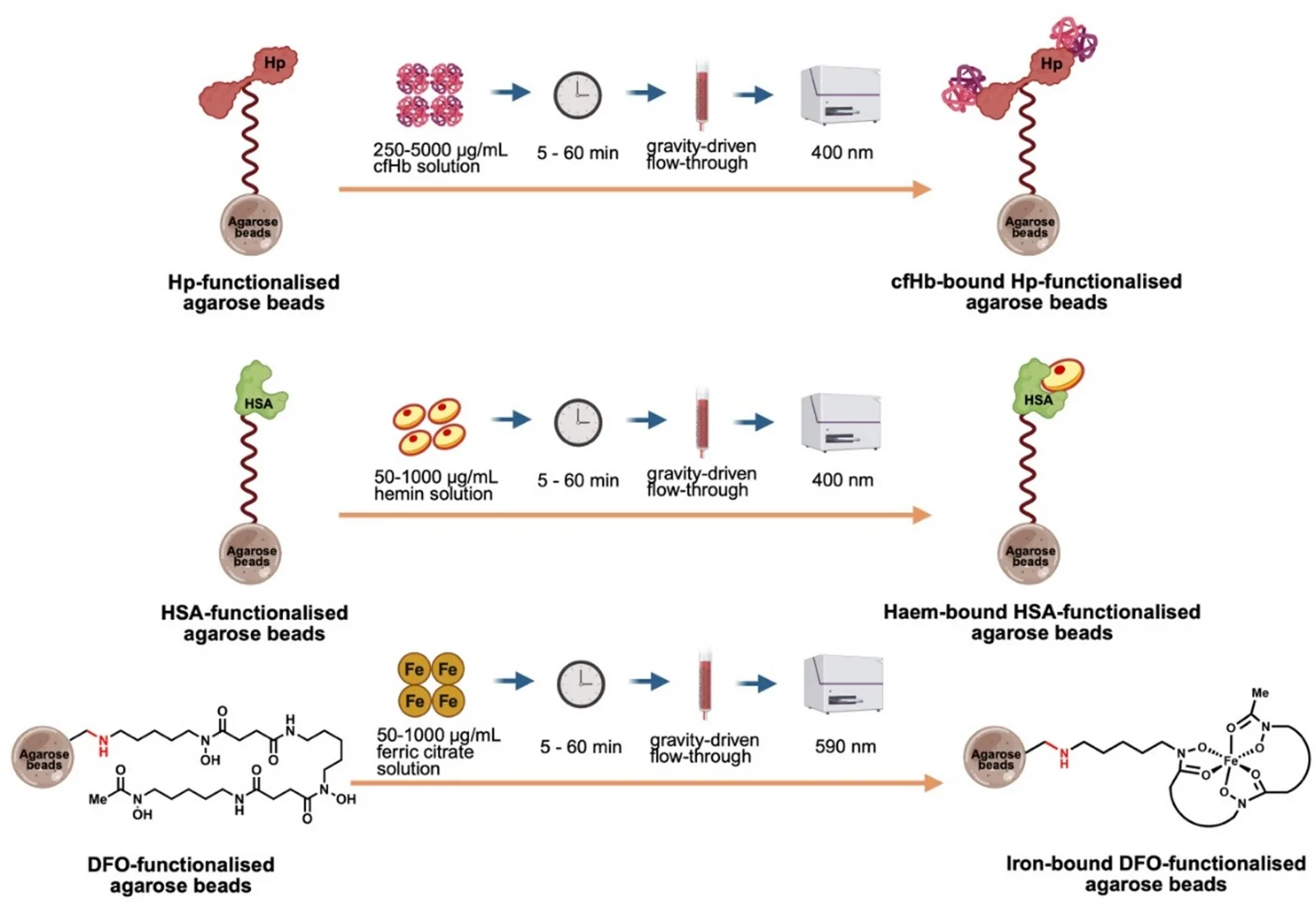
Schematic overview of affinity-functionalized agarose beads for the selective capture and quantification of hemolysis-associated byproducts. Hp-, HSA-, and DFO-functionalized beads were used to bind cfHb, haem, and iron, respectively, with target depletion quantified by colorimetric absorbance assays.

A series of experiments were conducted to determine the maximum binding capacity of the functionalized beads for their respective hemolysis byproducts (Fig. 3).

#### Preparation of Hb, haem, and iron solutions

Lyophilized human Hb was dissolved in phosphate-buffered saline (PBS, pH 7.4) to prepare solutions ranging from 250 to 5000 µg/mL, filtered through a 0.4 μm syringe filter, and freshly prepared for each experiment.

Porcine-derived hemin was first dissolved in 0.1 M NaOH and diluted 1:9 in PBS to 50–1000 µg/mL, filtered (0.4 μm), and freshly prepared on the day of use.

Ferric citrate was dissolved in PBS (pH 7.4) to prepare a 1000 µg/mL stock solution, stirred at 500 rpm for 2 h at 40 °C to ensure full dissolution, and diluted to 50–1000 µg/mL.

#### Binding efficiency studies

Three sets of experiments were conducted (*n* = 4 for each condition).

Time-course study: In separate packed columns, 0.5 mL of Hp-, HSA-, and DFO-functionalized beads (Hp LL = 74.4 mg/mL, HSA LL = 84.6 mg/mL, DFO LL = 43.3 mg/mL) were incubated with 2 mL of Hb (5000 µg/mL), hemin (1000 µg/mL), or ferric citrate (1000 µg/mL) solutions, respectively, for 5, 15, 30, and 60 min.

Effect of the affinity ligand LL: Beads with varying LLs (Hp: 32.7–74.4 mg/mL; HSA: 51.4 − 84.6 mg/mL; DFO: 8.9–43.3 mg/mL) were incubated with solutions of Hb (5000 µg/mL, 77.5 µM), hemin (1000 µg/mL, 1.5 mM), and ferric citrate (1000 µg/mL, 4 mM), respectively, for 30 min.

Effect of hemolysis byproduct concentration: In separate columns, 0.5 mL of the functionalized beads with the lowest LLs (Hp: 32.7 mg/mL, 0.3 µmol/mL; HSA: 51.4 mg/mL, 0.8 µmol/mL; DFO: 8.9 mg/mL, 13.6 µmol/mL) were incubated with 2 mL of Hb (250–5000 µg/mL), hemin (50–1000 µg/mL), or ferric citrate (50–1000 µg/mL) for 30 min.

After incubation, the solutions were drained under gravity, the beads were washed with 4 mL of PBS (pH 7.4) for 5 min, and the filtrates were used to calculate the binding efficiency. Unmodified beads served as controls.

### Binding of hemolysis byproducts in blood plasma and whole blood

#### Plasma isolation and spiking

Plasma was isolated from fresh leukodepleted clinical blood by centrifugation at 2000 g for 10 min at RT, aliquoted, and stored at − 20 °C. Before the experiments, the plasma was thawed at RT. Hb, hemin, and ferric citrate were dissolved in plasma to achieve final concentrations of 250–5000 µg/mL (Hb), 50–1000 µg/mL (hemin), and 50–1000 µg/mL (iron). Hemin was first dissolved in 0.1 M NaOH and then added to the plasma, and the resulting hemin-spiked plasma was neutralized to pH 7.4 using 1 M hydrochloric acid (HCl).

#### Preparation of hemolyzed whole blood

Whole blood was frozen at − 80 °C for 2 h to induce hemolysis, thawed at RT, and centrifuged at 2000 g for 10 min to isolate plasma containing free Hb, haem, and iron. The hemolyzed plasma was then added to fresh whole blood to obtain final hemolysis products in the following ranges: fHb, 250–5000 µg/mL; free haem, 50–1000 µg/mL; and free iron, 50–1000 µg/mL.

#### Binding efficiency studies

2 mL of spiked plasma or hemolyzed whole blood was incubated with 0.5 mL of Hp-, HSA-, or DFO-functionalized beads (LLs: Hp = 32.7 mg/mL; HSA = 51.4 mg/mL; DFO = 8.9 mg/mL) for 30 min at RT (*n* = 4). After incubation, the unbound Hb, haem, and iron were eluted, the beads were washed with 4 mL of PBS (pH 7.4), and the filtrates were analyzed to determine the binding levels of Hb, haem, and iron. Control experiments were conducted using unmodified beads.

### Quantification of hemolysis byproducts

The binding efficiencies of cfHb,haem and iron to the functionalized beads were quantified via QuantiChrom™ Hb, haem, and iron assay kits following the manufacturer’s instructions. For all the assays, the filtrates from the binding experiments were analyzed via a Clariostar Plus microplate reader. The absorbance was measured at 400 nm for cfHb andhaem and at 590 nm for iron. Concentrations were calculated via appropriate calibrators or standard curves generated with each kit. The amount of each analyte bound to the beads was determined by subtracting the filtrate concentration from the initial concentration of the stock solution.

### Column scale-up study

A 250 mL batch of Hp-, HSA-, and DFO-functionalized beads (90:5:5 ratio) was packed into a 600 mL filtration column and incubated with 200 mL of hemolyzed whole blood (cfHb = 5000 µg/mL, haem = 1000 µg/mL, iron = 1000 µg/mL) for 30 min (*n* = 4). The blood was drained by gravity to model the intended cardiotomy/suctioned-blood pathway configuration, and the beads were washed with 200 mL of PBS. The filtrates were analyzed for cfHb, haem, and iron contents via the QuantiChrom assay. Unmodified beads were used as controls.

The experimental conditions used in this study were interpreted using empty-bed contact time (EBCT) as a standard packed-bed parameter, as described in the Supplementary Materials**.** This framework was used to relate column performance to residence time in the intended gravity-fed configuration and to support scale-up interpretation.

### Statistical analysis

The data were analyzed *via* GraphPad Prism 10 (GraphPad Software, USA). All experiments were performed with a minimum of three independent replicates, unless otherwise stated. The data are presented as the means ± standard deviations (SDs), with individual data points shown where appropriate. For concentration-dependent immobilization and binding studies, presented as XY plots, the trends were assessed via regression analysis. When statistical comparisons were performed, one-way ANOVA was used to evaluate differences within the functionalized bead groups only (e.g., across ligand loading levels, incubation times, or analyte concentrations), followed by Holm–Šidák post hoc tests where applicable. Two-way ANOVA was used, where appropriate, to assess the effects of multiple factors (e.g., storage conditions and time; Supplementary data). Statistical significance was defined as *P* < 0.05.

## Results

### Affinity ligand immobilization

#### Qualitative characterization

**Fig. 4:**
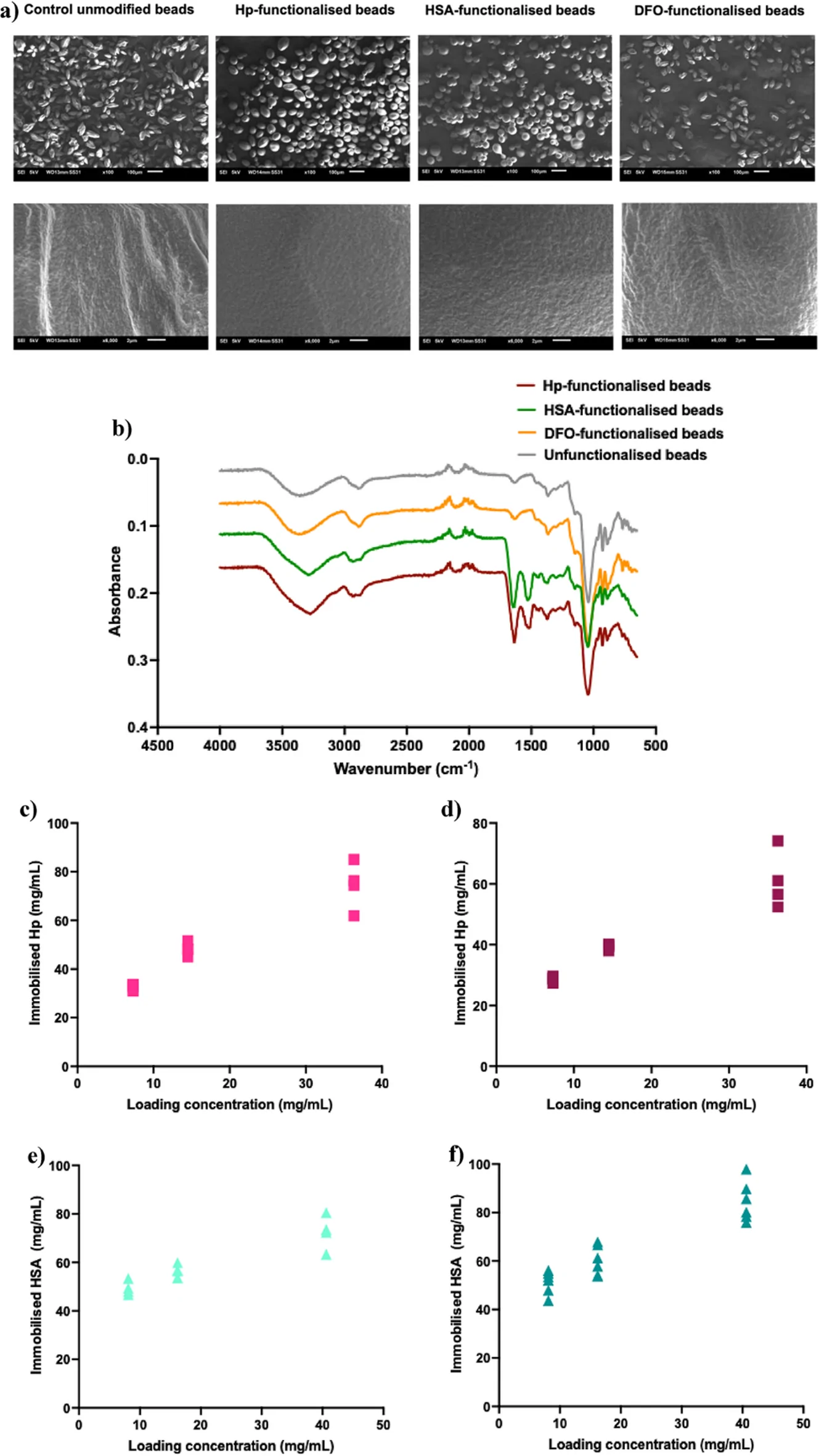

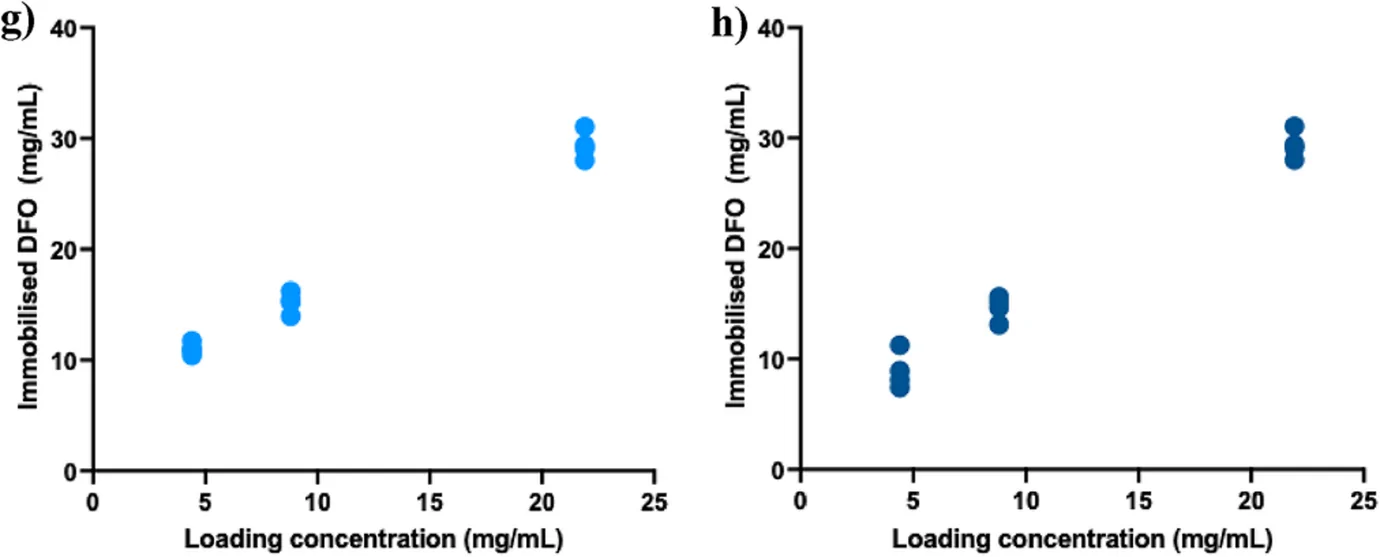
**(a)** SEM images of unmodified and affinity ligand-functionalized glyoxal agarose beads. Top panels: 100 μm scale (×100 magnification); bottom panels: 2 μm scale (×6 K magnification). **(b)** FTIR spectra of unmodified beads (gray) and beads functionalized with Hp (red), HSA (green), and DFO (orange). **(c–h)** Quantitative immobilization of affinity ligands as a function of input concentration: Hp was measured by the Pierce 660 nm assay **(c)** and BCA assay **(d)**; HSA was measured by the Pierce 660 nm assay **(e)** and BCA assay **(f)**; DFO was measured by the OPA assay **(g)** and spectrophotometric assay at 430 nm **(h)**. The data are shown as individual data points (*n* = 4 per condition). Lines represent regression fits used to assess concentration-dependent trends.

The surface morphology of the glyoxal agarose beads before and after affinity-ligand immobilization is shown in Fig. 4a. Scanning electron microscopy (SEM) images of the unmodified beads revealed a rough, granular surface with elongated, wrinkled structures, likely resulting from freeze-drying. Upon immobilization with Hp or HSA, the beads appeared smoother and more rounded, with reduced wrinkling, consistent with protein-coating masking of surface irregularities. In contrast, the DFO-functionalized beads showed no marked morphological changes compared with the unmodified controls, possibly because of the smaller molecular size of DFO, which may not significantly affect the surface topography. Fourier **t**ransform **i**nfra**r**ed (FTIR) analysis (Fig. 4b) confirmed successful ligand immobilization. The unmodified beads exhibited O–H stretching (~ 3400 cm⁻¹) and polysaccharide backbone vibrations (~ 1100 cm⁻¹). After Hp or HSA coupling, additional peaks corresponding to amide I (~ 1650 cm⁻¹) and amide II (~ 1550 cm⁻¹) appeared, indicating covalent protein attachment via peptide backbones. Broadening of the 3400 cm⁻¹ band also suggested N–H stretching from the protein moieties. DFO-functionalized beads showed only minor spectroscopic changes, with weak peaks between 1600 and 1500 cm⁻¹, suggesting lower surface coverage.

#### Quantitative characterization

The immobilization efficiency of each affinity ligand was optimized by varying the ligand input concentration. For Hp-functionalized glyoxal agarose beads (Fig. 4c and d), LL increased with increasing Hp concentration over the range of 7.3–36.3 mg/mL (0.07–0.36 µmol/mL, based on an average MW of 100 kDa). Regression analysis revealed a strong concentration-dependent increase in LL, with correlation coefficients of *r* = 0.91 (Pierce 660 nm assay) and *r* = 0.88 (BCA assay), which was consistent with increasing surface occupancy and a tendency toward saturation at higher input concentrations. At 36.3 mg/mL, an LL of 74.4 ± 11.7 mg/mL (0.7 µmol/mL) was achieved via the Pierce 660 nm assay, corresponding to an immobilization efficiency of 25.7 ± 4.0%. The BCA assay yielded an LL of 61.0 ± 11.5 mg/mL (0.6 µmol/mL) and an efficiency of 21.0 ± 3.9%.

A similar concentration-dependent trend was observed for the HSA-functionalized beads (Fig. 4e and f). Increasing the HSA input concentration from 8.1 to 40.6 mg/mL (0.12–0.61 µmol/mL) resulted in progressively higher LLs, with regression analysis indicating a strong positive relationship between the input concentration and ligand loading (*r* = 0.85 for the Pierce 660 nm and the BCA assays). The Pierce 660 nm assay yielded an LL of 72.5 ± 8.6 mg/mL (1.1 µmol/mL) and an immobilization efficiency of 22.3 ± 2.7%, whereas the BCA assay yielded an LL of 84.6 ± 8.2 mg/mL (1.2 µmol/mL) and an efficiency of 21.1 ± 0.9%.

For DFO-functionalized beads, LL increased proportionally with increasing input concentration over the range of 4.4–21.9 mg/mL (6.7–33.3 µmol/mL) (Fig. 4g and h). Linear regression analysis revealed an approximately linear relationship across the tested range, with high correlation coefficients (*r* = 0.98 for the OPA assay and *r* = 0.99 for the 430 nm spectrophotometric assay), suggesting no evidence of saturation within the tested loading range. At 21.9 mg/mL, the OPA assay resulted in an LL of 43.3 ± 4.8 mg/mL (65.9 ± 7.4 µmol/mL), corresponding to an immobilization efficiency of 24.7 ± 2.8%. The 430 nm assay yielded an LL of 29.4 ± 1.5 mg/mL (44.7 ± 2.3 µmol/mL), corresponding to an efficiency of 16.9 ± 0.8%.

### Binding efficiency of affinity ligand-functionalized agarose beads

#### Hemoglobin binding

**Fig. 5:**
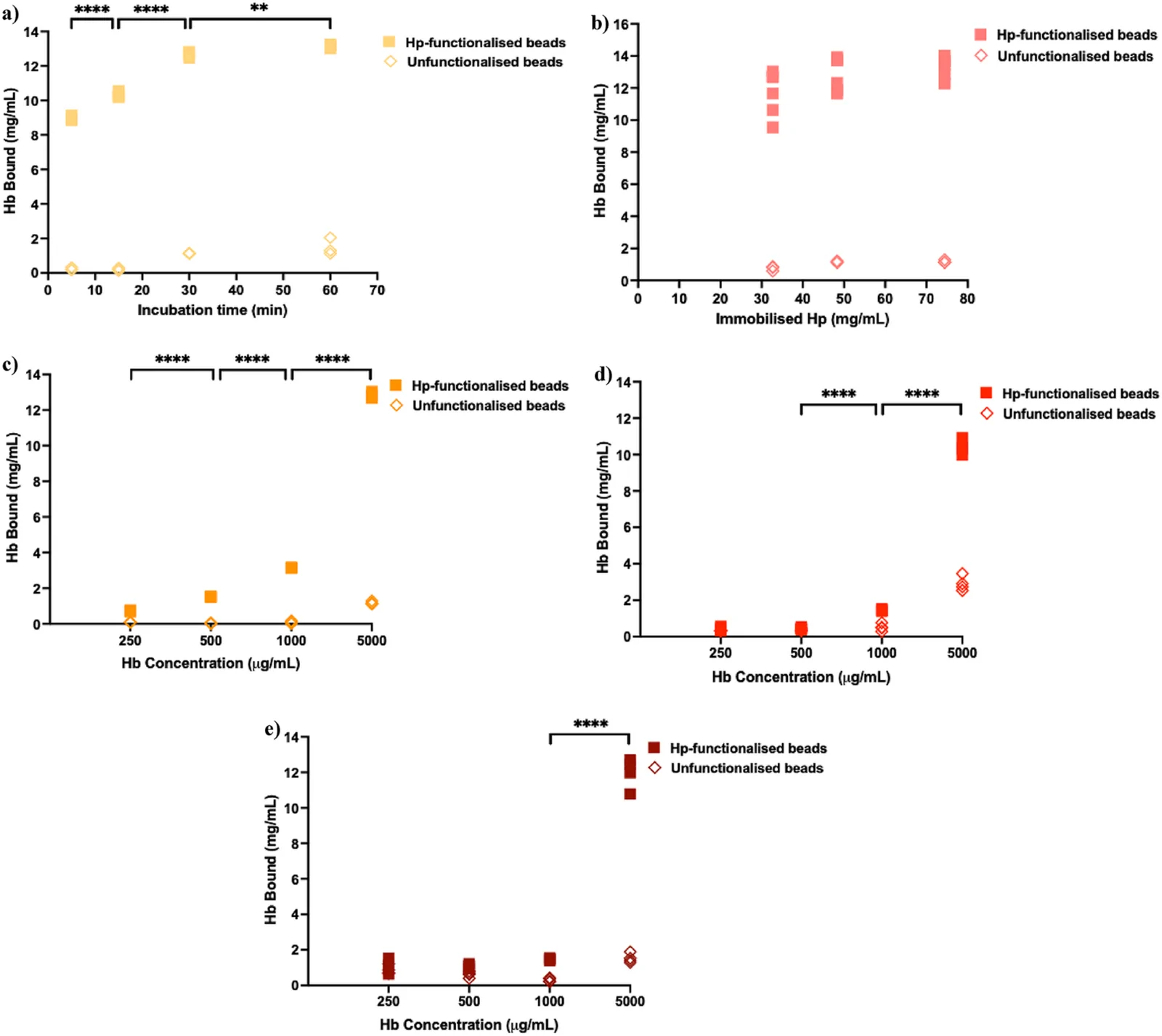
Evaluation of Hb binding by Hp-functionalized glyoxal agarose beads measured via the Quantichrom Hb assay. **(a)** Effect of incubation time (5–60 min) on Hb binding from an aqueous buffered Hb solution (PBS, pH 7.4). **(b)** Effect of the Hp ligand LL (32.7–74.4 mg/mL) on Hb binding from aqueous buffer. **(c)** Effect of the Hb concentration (250–5000 µg/mL) on Hb binding from aqueous buffer. **(d)** Hb binding from human plasma spiked with Hb (250–5000 µg/mL). **(e)** Hb binding from hemolyzed whole blood containing Hb (250–5000 µg/mL). The data are shown as individual data points (*n* = 4). Where indicated, statistical analysis was performed between Hp-functionalized bead groups only via one-way ANOVA; significance levels are denoted as **P ≤ 0.01 and ****P ≤ 0.0001.

In aqueous buffer solution: The Hb binding performance of Hp-functionalized glyoxal agarose beads was evaluated as a function of incubation time, Hb concentration, and ligand LL. For time-course studies, beads with an LL of 74.4 ± 11.7 mg/mL (0.7 ± 0.1 µmol/mL) were incubated with Hb (5 mg/mL, 77.5 µM) in PBS (pH 7.4) for up to 60 min (Fig. 5a). Hb binding increased over time, with the majority of binding occurring within the first 30 min (12.7 ± 0.2 mg/mL, *P* ≤ 0.0001), after which no further appreciable increase was observed, indicating that binding equilibrium was reached within this timeframe. The influence of LL on Hb binding was assessed using beads functionalized with Hp loadings ranging from 32.7 to 74.4 mg/mL (Fig. 5b). Across this range, Hb binding by the functionalized beads remained relatively constant at approximately 12–13 mg/mL, suggesting that binding saturation occurred within the tested range. One-way ANOVA performed on the functionalized bead groups confirmed that there were no statistically significant differences in Hb binding across the LL conditions (*P* > 0.05). Concentration-dependent Hb binding was investigated by incubating Hp-functionalized beads (LL: 32.7 ± 1.5 mg/mL) with increasing Hb concentrations (250–5000 µg/mL) (Fig. 5c). Hb binding progressively increased with increasing Hb concentration, which is consistent with a concentration-driven binding process. At higher Hb concentrations, binding approached a plateau, indicating saturation of the available Hp-binding sites. At all the tested concentrations, the functionalized beads exhibited substantially higher Hb binding than the unmodified controls did, indicating minimal nonspecific adsorption.

In human plasma: The Hb-binding capacity of Hp-functionalized beads was further evaluated in human plasma spiked with increasing concentrations of Hb (250–5000 µg/mL) (Fig. 5d). At lower Hb concentrations (250–500 µg/mL), Hb binding by the functionalized beads was comparable to that of the unmodified beads. However, at higher Hb concentrations, the Hp-functionalized beads demonstrated markedly increased binding. At 1000 µg/mL Hb, the functionalized beads bound 1.4 ± 0.06 mg/mL Hb, whereas unmodified beads bound 0.5 ± 0.2 mg/mL Hb. At 5000 µg/mL, Hb binding by the functionalized beads increased to 10.4 ± 0.5 mg/mL, corresponding to a binding efficiency of 47.1 ± 2.1%. Statistical comparison via one-way ANOVA across functionalized bead conditions confirmed a significant increase in Hb binding with increasing Hb concentration (*P* ≤ 0.0001), which was consistent with the concentration-dependent trends observed in the buffer.

In hemolyzed whole blood: The performance of Hp-functionalized beads was assessed in hemolyzed whole blood containing increasing Hb concentrations (250–5000 µg/mL) (Fig. 5e). Similar to plasma, Hb binding at lower concentrations (250–500 µg/mL) was limited and comparable to that of unmodified beads. At 1000 µg/mL, the functionalized beads bound 1.5 ± 0.1 mg/mL Hb, increasing further to 11.9 ± 1.0 mg/mL at 5000 µg/mL, corresponding to a binding efficiency of 61.6 ± 5.3%. One-way ANOVA across the functionalized bead groups revealed a statistically significant effect of the Hb concentration on the binding capacity of hemolyzed whole blood (*P* ≤ 0.05), confirming that Hp-functionalized beads retained effective Hb binding in complex biological matrices.

#### Haem binding

**Fig. 6:**
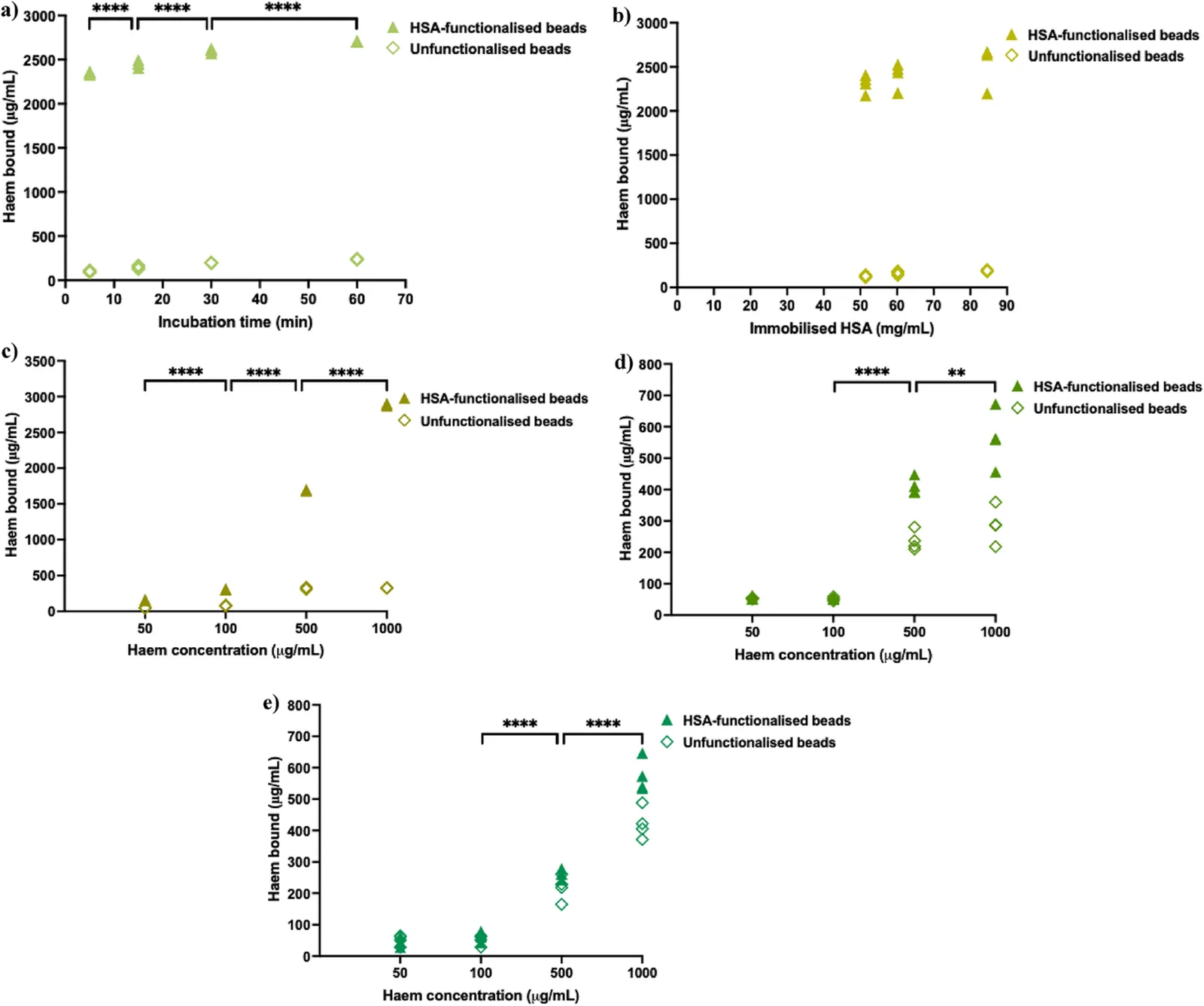
Evaluation of haem binding by HSA-functionalized glyoxal agarose beads measured via the Quantichrom haem assay. **(a)** Effect of incubation time (5–60 min) on haem binding from an aqueous buffered haem solution (PBS, pH 7.4). **(b)** Effect of the HSA ligand LL (51.4–84.6 mg/mL) on haem binding from aqueous buffer. **(c)** Effect of the haem concentration (50–1000 µg/mL) on haem binding from aqueous buffer. **(d)** Haem binding from human plasma spiked with haem (50–1000 µg/mL). **(e)** Haem binding from hemolyzed whole blood containing haem (50–1000 µg/mL). The data are shown as individual data points (*n* = 4). Where indicated, statistical analysis was performed between HSA-functionalized bead groups only via one-way ANOVA; significance levels are denoted as **P ≤ 0.01 and ****P ≤ 0.0001.

In aqueous buffer solution: The haem-binding performance of HSA-functionalized glyoxal agarose beads was evaluated as a function of incubation time, hemin concentration, and ligand LL. Beads with an LL of 84.6 ± 8.2 mg/mL (1.2 µmol/mL) showed a time-dependent increase in haem binding when incubated with hemin (1 mg/mL, PBS, pH 7.4), approaching equilibrium by 60 min (Fig. 6a). Across the tested range of HSA LLs (51.4–84.6 mg/mL), haem binding by the functionalized beads remained comparable, with one-way ANOVA confirming that LL had no significant effect on the binding capacity (*P* > 0.05) (Fig. 6b). Increasing the hemin concentration (50–1000 µg/mL) resulted in progressively increased haem binding by the HSA-functionalized beads (Fig. 6c). One-way ANOVA across the functionalized bead groups revealed a significant effect of the hemin concentration on binding (*P* ≤ 0.0001), which was consistent with the concentration-dependent binding behavior. At all the concentrations tested, the functionalized beads exhibited substantially higher haem binding than the unmodified controls did, indicating minimal nonspecific adsorption. At 50 µg/mL hemin, the HSA-functionalized beads captured 159.4 ± 1.6 µg/mL (76.4 ± 0.7%), whereas the control beads captured 47.7 ± 5.3 µg/mL (22.9 ± 2.6%). At higher concentrations of 100, 500, and 1000 µg/mL, the functionalized beads presented binding levels of 306.7 ± 6.4 µg/mL (76.0 ± 1.6%), 1693.3 ± 8.2 µg/mL (85.3 ± 0.4%), and 2624.4 ± 15.8 µg/mL (83.2 ± 0.5%), respectively.

In human plasma: HSA-functionalized beads (LL: 51.4 ± 4.7 mg/mL) were incubated with plasma spiked with hemin (50–1000 µg/mL) (Fig. 6d). At 50 and 100 µg/mL, binding was comparable between the functionalized and unmodified beads. Specifically, at 50 µg/mL, haem binding was 55.6 ± 6.0 µg/mL for the functionalized beads and 53.3 ± 2.0 µg/mL for the controls; at 100 µg/mL, the values were 52.1 ± 1.8 µg/mL and 52.0 ± 6.8 µg/mL, respectively. At 500 µg/mL, the functionalized beads captured 410.9 ± 31.8 µg/mL, whereas the control unmodified beads captured 236.7 ± 38.2 µg/mL. At 1000 µg/mL, the functionalized beads reached 562.6 ± 108.1 µg/mL, whereas the control beads reached 288.0 ± 71.0 µg/mL, demonstrating preserved haem-binding capacity at elevated concentrations despite the presence of plasma proteins. Statistical analysis restricted to the functionalized bead groups confirmed a significant concentration-dependent increase in haem binding (*P* ≤ 0.01).

In hemolyzed whole blood: Similar concentration-dependent trend was observed in hemolyzed whole blood (Fig. 6e). At lower haem concentrations (≤ 500 µg/mL), binding remained limited, whereas at 1000 µg/mL, HSA-functionalized beads achieved substantially greater haem binding (573.2 ± 63.3 µg/mL, 0.9 ± 0.1 µmol/mL; 16.4 ± 1.8%) than unmodified controls did (421.4 ± 60.1 µg/mL, 0.6 ± 0.1 µmol/mL; 12.1 ± 1.7%). One-way ANOVA across the functionalized bead groups confirmed the significant effect of the haem concentration on the binding capacity (*P* ≤ 0.0001), demonstrating preserved haem-binding performance under physiologically complex conditions.

#### Iron binding

**Fig. 7:**
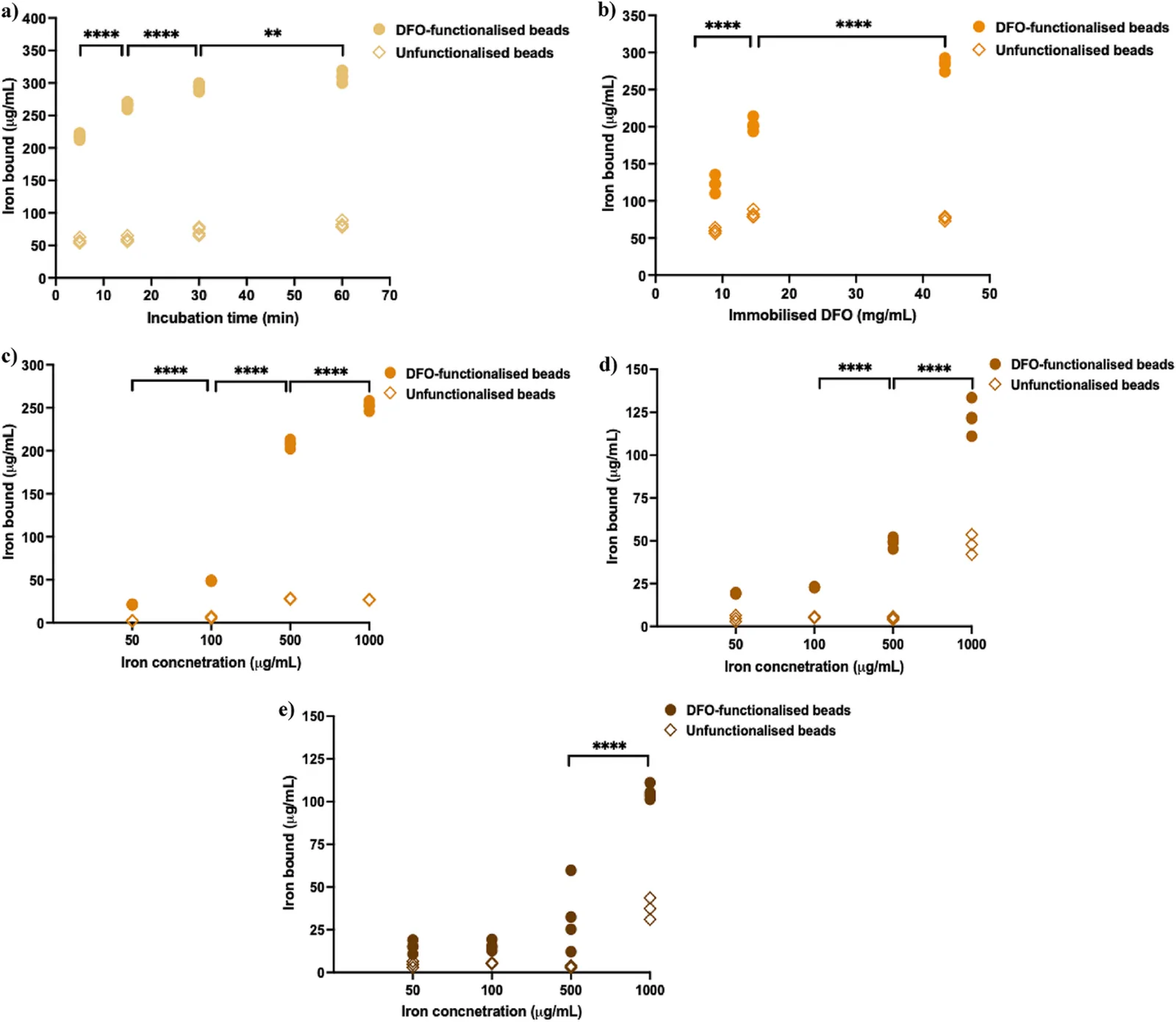
Evaluation of the iron binding efficiency of DFO-functionalized glyoxal agarose beads as measured by the Quantichrom iron assay. **(a)** Effect of incubation time (5–60 min) on iron binding from aqueous buffered iron solution (PBS, pH 7.4). **(b)** Effects of the DFO ligand LL (8.9–43.3 mg/mL) on iron binding from aqueous buffer. **(c)** Effect of iron concentration (50–1000 µg/mL) on iron binding from aqueous buffer. **(d)** Iron binding efficiency of plasma spiked with iron (50–1000 µg/mL). **(e)** Iron binding efficiency from hemolyzed whole blood (50–1000 µg/mL iron). The data are shown as individual data points (*n* = 4). Where indicated, statistical analysis was performed between DFO-functionalized bead groups only via one-way ANOVA; significance levels are denoted as **P ≤ 0.01 and ****P ≤ 0.0001.

In aqueous buffer solution: The iron binding capacity of DFO-functionalized glyoxal agarose beads was evaluated as a function of incubation time, ferric citrate concentration, and ligand LL. Beads with an LL of 43.3 ± 4.8 mg/mL (65.9 ± 7.4 µmol/mL) showed a time-dependent increase in iron binding when incubated with ferric citrate (1 mg/mL, 4 mM, PBS, pH 7.4), approaching equilibrium by 30 min (309.7 ± 13.7 µg/mL (1.3 ± 0.1 µmol/mL, 47.5 ± 2.1%)), with only a marginal additional increase at 60 min (Fig. 7a). Increasing DFO LL (8.9–43.3 mg/mL) resulted in increased iron binding capacity, reaching 284.3 ± 9.3 µg/mL (1.2 ± 0.0 µmol/mL) at the highest loading, as determined by one-way ANOVA, confirming the significant effect of LL among the functionalized bead groups (*P* ≤ 0.0001) (Fig. 7b). Concentration-dependent binding was observed when the beads (LL: 8.9 ± 2.0 mg/mL) were incubated with increasing ferric citrate concentrations (50–1000 µg/mL) (Fig. 7c). Absolute iron binding increased with increasing iron concentration, whereas the binding efficiency decreased at higher iron loads. At 50 µg/mL, the beads bound 21.1 ± 0.1 µg/mL (0.3 ± 0.0 µmol/mL; 90.4 ± 0.4%), whereas at 1000 µg/mL, the binding increased to 252.3 ± 6.0 µg/mL (3.9 ± 0.1 µmol/mL), with a reduced efficiency (44.3 ± 1.1%).

In human plasma: In human plasma spiked with iron (50–1000 µg/mL), iron binding by DFO-functionalized beads increased with increasing iron concentration (Fig. 7d). At lower concentrations (50–100 µg/mL), the binding was comparable to that of the unmodified beads, whereas at 500 and 1000 µg/mL, the functionalized beads demonstrated markedly greater iron binding. Binding increased from 49.0 ± 5.5 µg/mL (21.5 ± 2.4%) at 500 µg/mL to 122.0 ± 11.3 µg/mL (23.8 ± 4.4%) at 1000 µg/mL. One-way ANOVA restricted to the functionalized bead groups confirmed a significant concentration-dependent effect on iron binding (*P* ≤ 0.0001).

In hemolyzed whole blood: A similar trend was observed in hemolyzed whole blood (Fig. 7e). At iron concentrations ≤ 500 µg/mL, binding remained limited and comparable between the bead types. At 1000 µg/mL, the DFO-functionalized beads achieved substantially greater iron binding than the unmodified controls did. Statistical analysis across the functionalized bead groups confirmed the significant effect of the iron concentration on the binding capacity (*P* ≤ 0.0001), demonstrating effective iron sequestration under physiologically complex conditions.

Adsorption behavior: The concentration-dependent binding profiles showed a tendency toward saturation at higher analyte concentrations, consistent with finite ligand-binding capacity and qualitatively consistent with Langmuir-type adsorption behavior. The apparent maximum binding values were approximately 13 mg/mL for cfHb, 2.6 mg/mL for haem, and 0.3 mg/mL for iron, consistent with the experimental plateaus.

### Column scale-up study

**Fig. 8:**
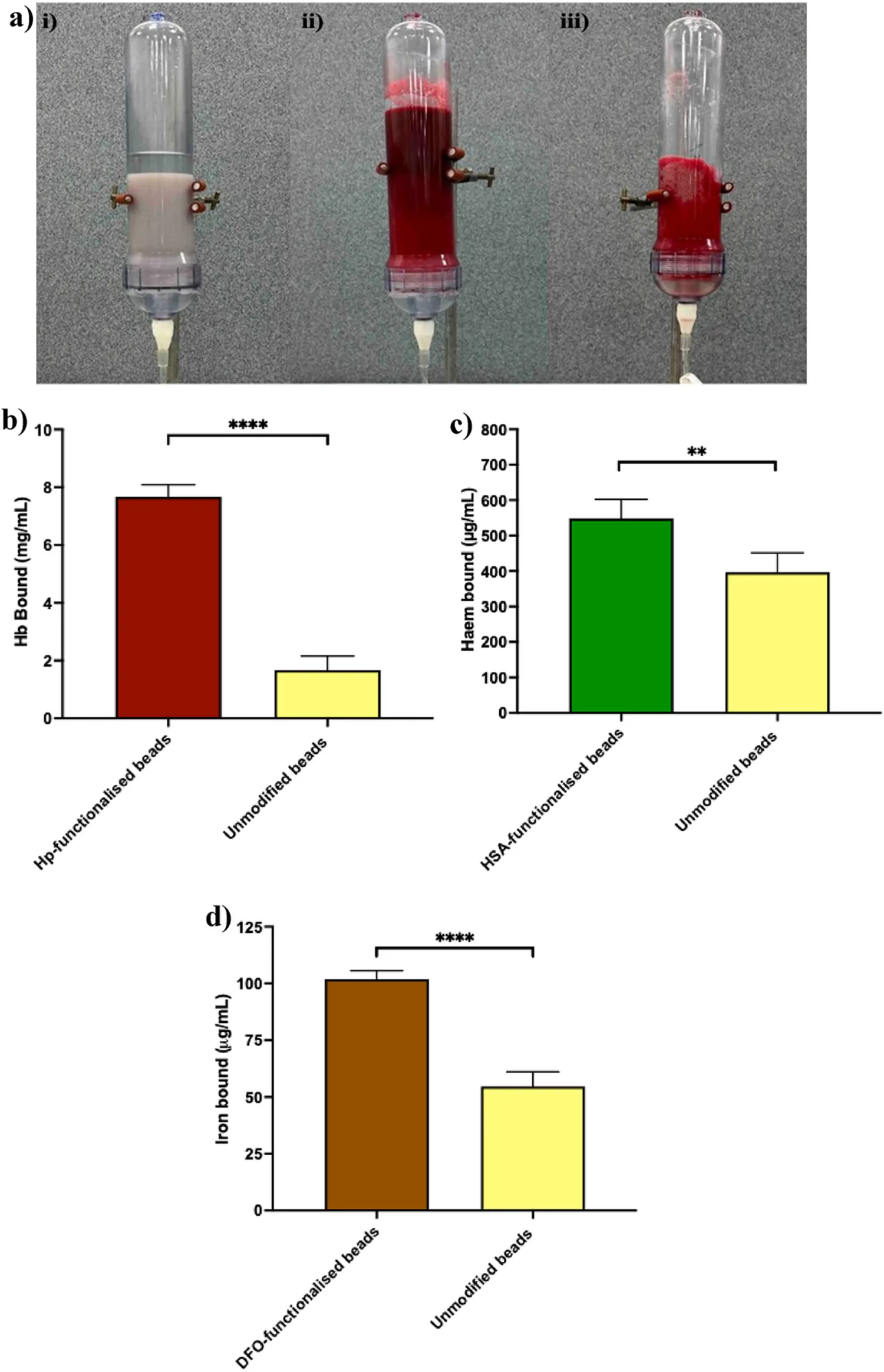
Scale-up study evaluating the Hb, haem, and iron binding efficiency of functionalized glyoxal agarose beads. **(a**) Images of the hemolysis filter: **(i)** 250 mL of functionalized beads packed into the filter column prior to treatment, **(ii)** 200 mL of hemolyzed blood passed through the filter under gravity, and **(iii)** the filter column was washed with 200 mL of PBS (pH 7.4). **(b)** Hb binding efficiency of Hp-functionalized beads measured by the Quantichrom Hb assay. **(c)** Haem binding efficiency of HSA-functionalized beads measured by the Quantichrom haem assay. **(d)** Iron binding efficiency of DFO-functionalized beads measured by the Quantichrom iron assay. The data are the means ± SDs (*n* = 4). Statistical significance: *P ≤ 0.05; ***P ≤ 0.001; ****P ≤ 0.0001; Student’s *t* test.

The performance of Hp-, HSA-, and DFO-functionalized glyoxal agarose beads was evaluated in a scaled-up gravity-fed column containing 250 mL of beads mixed at a 90:5:5 ratio (Hp: HSA: DFO). The column was incubated with 200 mL of hemolyzed whole blood containing cfHb (5000 µg/mL), haem (1000 µg/mL), and iron (1000 µg/mL) for 30 min (Fig. 8). Based on the processed volume and experimental duration, this corresponded to an approximate flow rate of 6.7 mL/min and an estimated EBCT of approximately 35–40 min.

This experiment simultaneously tested all three analytes, allowing evaluation of target removal under multicomponent conditions. Relative to unmodified controls, the functionalized column achieved markedly greater removal: cfHb 7.7 ± 0.5 mg/mL vs. 1.7 ± 0.6 mg/mL (*P* ≤ 0.001); haem 548.5 ± 65.9 µg/mL vs. 396.7 ± 66.8 µg/mL (*P* ≤ 0.05); and iron 102.0 ± 4.5 µg/mL vs. 54.6 ± 7.8 µg/mL (*P* ≤ 0.001). These findings indicate that the mixed functionalized bead column retained target-removal performance under multicomponent whole-blood conditions. Comparable binding performance across scales suggests that adsorption performance is influenced by residence time, supporting scale-up based on preservation of key column parameters.

## Discussion

Hemolysis remains a significant complication in extracorporeal circuits, such as CPB, ECMO, dialysis, and blood salvage procedures. The release of RBC contents, specifically cfHb, haem, and redox-active iron, can rapidly overwhelm endogenous scavenging systems, leading to oxidative stress, vascular dysfunction, inflammation, and organ injury^39–46^. While current interventions are largely supportive, no clinically available devices selectively and simultaneously remove these toxic hemolytic byproducts from suctioned or processed blood before return to the extracorporeal main circuit.

This study presents the development of a novel “triple filter” designed for use in extracorporeal circuit, employing glyoxal agarose beads functionalized with Hp, HSA, and DFO to sequester cfHb, haem, and iron. In recent years, additional adsorption technologies, such as CytoSorb^®^ cartridges, have been used in ECMO and continuous renal replacement therapy (CRRT) settings to remove inflammatory mediators and some circulating toxins. However, they are typically nonselective and may inadvertently deplete beneficial plasma components or interfere with therapeutic drug levels^25,47–49^. Dialysis membranes with modified permeability have shown modest cfHb removal depending on pore size but lack target specificity and are not designed for the simultaneous removal of multiple hemolysis byproducts^23,24^. Additionally, existing iron chelation therapies, such as intravenous desferrioxamine, are systemic and unsuitable for localized extracorporeal applications^29,30^. In contrast, the present affinity-based strategy enables selective and simultaneous capture of the three major hemolytic byproducts through immobilized ligand-specific interactions, offering a distinct mechanistic and translational advantage. Thus, this platform represents a functionally specific and potentially clinically relevant addition to current extracorporeal detoxification strategies^50–52^.

The use of glyoxal agarose as a support matrix is predicated on its biocompatibility, mechanical stability, and high porosity, which support efficient ligand coupling and effective target accessibility^34–36^. Optimization studies identified glyoxal-functionalized agarose as a superior matrix compared with NHS-ester- and CNBr-activated supports (data not shown), providing greater ligand immobilization across a range of reaction conditions. The ε-amino groups of lysine residues in Hp and HSA underwent Schiff base formation with aldehyde groups on the beads, followed by reductive amination to yield stable covalent linkages. Similarly, DFO was immobilized via its primary amine groups. Covalent attachment ensures ligand stability, which is essential for storage, reuse, and safe clinical application^36^.

Surface and spectroscopic characterization confirmed successful ligand immobilization. SEM revealed distinct morphological changes following Hp and HSA functionalization (Fig. 4a), whereas DFO-functionalized beads exhibited minimal surface alteration, likely due to DFO’s small size and limited surface coverage. FTIR spectroscopy further confirmed the successful immobilization of Hp and HSA on glyoxal agarose beads, as evidenced by the appearance of the characteristic amide I and amide II peaks (Fig. 4b). However, the DFO-functionalized beads exhibited only minor spectral changes, indicating potential challenges in characterizing the immobilization of small molecules compared with that of larger proteins. Further optimization studies demonstrated that increasing the ligand concentration during coupling increased the total loading but reduced the immobilization efficiency, likely because of steric hindrance and surface crowding (Fig. 4c and h). Such trade-offs are typical for high-density functionalization and can be mitigated through process strategies such as volume minimization and reagent recycling^34–36^.

The safety and longevity of biomedical filtration devices significantly depend on the stability of their active components. Uncontrolled leaching, degradation, or loss of immobilized affinity ligands during storage or use can compromise therapeutic functionality^36^. Over a 60-day storage period under refrigerated and frozen conditions, all the functionalized beads exhibited minimal ligand leaching and retained consistent binding activity (Table S1 and Figure S1). These results confirm the structural integrity and bioactivity of the immobilized ligands, underscoring the potential of the device for extended storage and use in clinical settings.

The interaction of biomaterials with the blood may result in an unwanted inflammatory response and the activation of coagulation, as occurs with ECC^53^. To evaluate hemocompatibility, we assessed the thrombin–antithrombin (TAT) complex and D-dimer formation following the exposure of whole blood from healthy volunteers to functionalized and unmodified beads (Figure S2). These markers reflect coagulation and fibrinolysis, respectively. No significant increase in either parameter was observed, indicating that the functionalized beads did not trigger a procoagulant response under the tested conditions. While these findings are encouraging, a more comprehensive hemocompatibility assessment, including platelet activation, complement activation, and hemolysis markers, will be required for full clinical validation and will be addressed in future studies.

Optimizing the binding efficiency of functionalized beads across different media, buffered solutions, plasma, and whole blood, requires a thorough understanding of the parameters governing bead–target interactions. In buffered aqueous systems, where interference is minimal, variables such as incubation time, ligand loading density, and target concentration primarily dictate binding performance. The concentration-dependent binding profiles showed saturation behaviour consistent with finite ligand-binding capacity. Under these controlled conditions, the affinity ligands interact efficiently with their respective targets. Binding studies in buffered systems demonstrated high removal efficiencies (~ 80%) for cfHb (Fig. 5a and c), haem (Fig. 6a and c), and iron (Fig. 7a and c). Under these simplified conditions, variables such as the incubation time, ligand density, and target concentration predominantly dictated the binding outcomes. Saturation effects were observed at high ligand loadings for Hp and HSA, likely owing to steric hindrance and suboptimal ligand orientation on the bead surfaces. DFO-functionalized beads did not exhibit such limitations, with binding increasing proportionally with increasing ligand loading, which is consistent with the small size of DFO and the diffusion behavior of iron ions^52^. The observed time-dependent binding profiles indicate adsorption-driven interactions with finite binding capacity, with equilibrium typically reached within 30–60 min under the tested conditions.

The complexity of biological fluids, such as plasma and whole blood, introduces additional variables that can significantly affect bead performance. Plasma contains a multitude of endogenous proteins, including Hp, hemopexin, HSA, and transferrin, which can compete with functionalized beads for hemolysis byproducts^11–13^. At low concentrations of hemolytic byproducts, these endogenous proteins can effectively sequester Hb, haem, and iron before coming into contact with relevant functionalized beads. This competitive binding reduces the overall efficacy of the beads in plasma at low concentrations of hemolytic byproducts. Additionally, the presence of nonspecific plasma proteins may result in fouling of bead surfaces. Nonspecific adsorption can obscure functional binding sites and decrease the availability of ligands for specific interactions, thereby reducing the binding capacity^54^. In whole blood, this challenge is further compounded by the presence of cellular elements and shear forces. RBCs, white blood cells, and platelets can physically obstruct beads, limiting their contact with target molecules. Nevertheless, our data showed that the beads maintained a significant binding capacity in both plasma (Figs. 5d and 6d, and 7d) and blood (Figs. 5e and 6e, and 7e), especially under conditions of elevated hemolysis, suggesting that the system would be most effective in clinical scenarios where these byproducts are abundant.

The scale-up study provides further insight into the translational potential of the system^55^. This experiment was conducted using hemolyzed whole blood containing cfHb, haem, and iron simultaneously, The system can be interpreted as a packed-bed adsorption column, in which performance is influenced by residence time. The estimated EBCT of approximately 35–40 min in the scaled system was consistent with smaller-scale conditions, and comparable binding performance was maintained. These findings provide initial evidence that the three ligand-functionalized bead types can operate concurrently in a mixed system under the intended gravity-fed mode of use. However, further investigation of competitive binding dynamics, cross-binding, flow distribution, pressure drop, and transport phenomena will require controlled cardiotomy-line simulations with time-resolved outlet sampling.

The relative abundance of individual hemolytic byproducts dictates the optimal ratios of immobilized affinity ligands required for effective extracorporeal removal. During CPB and other high-shear extracorporeal therapies, circulating concentrations of cfHb, haem, and redox-active iron rise rapidly and heterogeneously, depending on the circuit design, duration of support, transfusion burden, and patient-specific susceptibility to hemolysis^56,57^. Across multiple clinical studies, cfHb concentrations during CPB are most commonly reported to be in the range of approximately 50–500 mg/dL, with peak values exceeding 1 g/L in cases of severe hemolysis or prolonged extracorporeal support^58,59^. Concurrently, free haem concentrations are typically reported in the low-to-mid micromolar range (approximately 5–50 µM), whereas labile or nontransferrin-bound plasma iron can increase from submicromolar baseline levels to values exceeding 10–15 µM under hemolytic stress^59–65^.

Using clinically realistic concentration ranges derived from multiple independent cohorts undergoing CPB, ECMO, trauma resuscitation, or massive transfusion, we estimated the relative binding demand for each affinity ligand within the triple-filter system. Given the predominance of cfHb in terms of absolute mass burden, the filter architecture was intentionally biased toward Hp-functionalized beads. On this basis, we estimated that a packed-bed filter volume of approximately 200–250 mL, predominantly composed of Hp-functionalized beads, would be sufficient to address acute hemolytic burden immediately following CPB or other high-risk extracorporeal interventions. This design rationale was evaluated in a gravity-fed scale-up study employing intimately mixed Hp: HSA: DFO beads at a volume ratio of 90:5:5 within a 250 mL column (Fig. 8). Under hemolytic conditions representative of those reported clinically, the functionalized column achieved greater than 70% cfHb removal while simultaneously capturing clinically relevant quantities of haem and iron, in marked contrast to unmodified bead controls. Importantly, the modular nature of this platform permits future adjustment of ligand ratios to accommodate different hemolytic profiles, circuit configurations, or therapeutic requirements during extracorporeal support.

It is important to note that the intended initial application of this system is not as an inline filter within the main pump-driven circuit, but as a gravity-fed filtration step in the cardiotomy/suctioned-blood pathway. In this proposed configuration, blood collected from the surgical field would pass under gravity assisted flow through the packed bed triple filter en route to the cardiotomy reservoir and thence periodic return to the main circuit. Future work will focus on controlled cardiotomy-line simulations in which EBCT, bed volume, bed depth, pressure drop, flow distribution, and repeated blood loading can be systematically evaluated^66^. In addition, preliminary in vivo studies evaluating the system under physiologically relevant extracorporeal conditions are ongoing and will be reported separately.

## Conclusion

This study presents the development of a triple-affinity hemolysis filter designed for use in extracorporeal circuits, achieved through the covalent immobilization of Hp, HSA, and DFO onto glyoxal-functionalized agarose beads. This platform enables the simultaneous and selective removal of the three principal hemolysis byproducts- cfHb, haem, and iron- with high binding efficiencies across buffered solutions, plasma, and hemolyzed whole blood. Under optimized conditions, binding capacities of approximately 12.7 mg/mL for cfHb, 2.7 mg/mL for haem, and 0.3 mg/mL for iron were achieved, and these performances were maintained at clinically relevant scales, with effective removal of all three targets demonstrated in a 250 mL column system. The functionalized beads also showed minimal ligand leaching and no detectable increase in TAT or D-dimer under the tested ex vivo conditions. Furthermore, scale-up studies indicated that adsorption performance is influenced by residence time, suggesting that the system can be translated to larger configurations through preservation of key column parameters. The modular and scalable nature of this affinity-based system, combined with its mechanism-specific targeting, positions it as a promising adjunct to current extracorporeal modalities for mitigating hemolysis-associated complications. While the present study establishes a strong proof-of-concept, further work is required to conduct comprehensive hemocompatibility assessments, and to validate efficacy in preclinical and disease-relevant hemolytic settings. These efforts will support the progression of this technology toward clinical translation in CPB and other extracorporeal therapies.

## Supplementary Information

Below is the link to the electronic supplementary material.

Supplementary Material 1

## Data Availability

All the data supporting the findings of this study are included in this article and its supplementary information. Additional raw data are available from the corresponding authors upon request.
