# Supplementary material for: Development of a hemolysis filter system for the selective removal of free hemoglobin, haem and iron from blood envisaged for use in extracorporeal circuits
Source: Sci Rep. 2026 Jul 1;16:29465. doi: 10.1038/s41598-026-57088-y (PMC13597300; doi:10.1038/s41598-026-57088-y)
Supplement: Supplementary file 1 [file 41598_2026_57088_MOESM1_ESM.docx]

**Supplementary Material**

**Development of a hemolysis filter system for the selective removal of free hemoglobin, heme and iron from blood envisaged for use in extracorporeal circuits**

*Noora Naghavi^1,2^, Lisa Barbaro^3,4^, Mark JD Griffiths^1,5^, Simon Davidson^6^, Daniel D Melley^5^, John O’Neil^5^, Karl Riley^1^, Richard W Issitt^8^, Gregory J Quinlan^1, *,🖂^, Nathan A Davies^7, *,🖂^, and Alan C. Spivey^2, *, 🖂^.*

^1^National Heart and Lung Institute (NHLI), Guy Scadding Building, Imperial College London, 70 Cale Street, London, SW3 6PT, United Kingdom

^2^Department of Chemistry, Molecular Science Research Hub (MSRH), Imperial College London, White City Campus, 82 Wood Lane, London, W12 0BZ, United Kingdom

^3^Warren Centre for Neuroscience Drug Discovery, Vanderbilt University, Nashville, Tennessee 37232, United States

^4^Department of Pharmacology, Vanderbilt University School of Medicine, Nashville, Tennessee 37232, United States

^5^St Bartholomew’s Hospital, West Smithfield, London, EC1A 7BE, United Kingdom

^6^Faculty of Medical Science, Rayne Institute, University College London, 5 University Street, London, WC1E 6JF, United Kingdom

^7^Institute for Liver and Digestive Health, UCL Meical School, Royal Free Campus, Roland Hill Street, London NW3 2PF, United Kingdom

^8^Department of Children’s Cardiovascular Disease, UCL Institute of Cardiovascular Science, Zayed Centre for Research into Rare Disease in Children, 20 Guilford Street, London WC1N 1DZ, United Kingdom

*	🖂email: [g.quinlan@imperial.ac.uk](mailto:g.quinlan@imperial.ac.uk); [nathan.davies@ucl.ac.uk](mailto:nathan.davies@ucl.ac.uk); [a.c.spivey@imperial.ac.uk](mailto:a.c.spivey@imperial.ac.uk)

**S1. Materials**

Aldehyde-derived agarose beads [high density (6%) glyoxal agarose beads, 90 µm average diameter, nominal loading level (LL) 40-60 µmol/mL gel] were purchased from Gold Biotechnology (GoldBio, St. Louis, MO, USA). Human-derived haptoglobin (Hp) (MW: 100–400 kDa) was kindly supplied by Kedrion Biopharma (Borehamwood, UK). Lyophilized human serum albumin (HSA) (MW: 66.5 KDa), lyophilized human-derived hemoglobin (Hb) (MW: 64.5 KDa), porcine derived hemin (MW: 651.94 Da), desferrioxamine mesylate salt (DFO.MeSO_3_H) (MW: 656.79 Da), phosphate-buffered saline (PBS), fluoraldehyde™ o-phthaldialdehyde reagent solution (OPA), sodium bicarbonate (NaHCO_3_), sodium carbonate (Na_2_CO_3_), sodium borohydride (NaBH_4_), sodium chloride (NaCl), hydrochloric acid (HCl), sodium hydroxide (NaOH), and ferric citrate were purchased from Sigma-Aldrich (Merck Life Science UK Limited, Gillingham, UK). The Pierce™ BCA Protein Assay Kit (Cat No: 23227) and Pierce™ 660 nm Protein Assay Kit (Cat No: 22660) were purchased from Thermo Fisher Scientific (Life Technologies Ltd., Inchinnan Business Park, Paisley, UK). The Quantichrom^TM^ Hb assay kit (ab272533), Quantichrom^TM^ heme assay kit (ab272534), human D-dimer ELISA assay kit (ab260076), and human thrombin-antithrombin complex (TAT) ELISA assay kit (ab108907) were purchased from Abcam (Cambridge, UK). The Quantichrom^TM^ Iron Assay Kit was purchased from CliniSciences ( Slough, UK). Leukodepleted clinical spec whole blood (500 mL blood bag) was purchased from NHS Blood and Transplant (NHS BT; Colindale, UK).

**S2. Methods**

**S2.1. Immobilization of affinity ligands onto aldehyde-derived agarose beads**

The following protocol describes the preparation of 1 mL of immobilized beads. The coupling reaction can be linearly scaled by adjusting the amounts of beads, buffers, and ligand solutions. A 2 mL volume of glyoxal agarose bead slurry (50% slurry, equivalent to 1 mL packed bed volume [PBV]) was transferred into a 10 mL gel holding column fitted with a polyethylene filter. The slurry was drained using a vacuum pump aspirator. The beads were washed with deionized water (10 × PBV) to remove preservatives, followed by phosphate-buffered saline (PBS, pH 7.4; 5 × PBV), and equilibrated in coupling buffer (0.1 M NaHCO₃, 0.5 M NaCl, pH 10.0; 1 × PBV). Each of the three affinity ligands was covalently immobilized *via* Schiff base linkages onto aldehyde-derived agarose beads.

Three different concentrations of each affinity ligand were prepared in coupling buffer:

- Hp: 7.3, 14.5, and 36.3 mg/mL (0.07, 0.14, and 0.36 µmol/mL, respectively)
- HSA: 8.1, 16.2, and 40.6 mg/mL (0.12, 0.24, and 0.61 µmol/mL, respectively)
- DFO: 4.4, 8.8, and 21.9 mg/mL (6.7, 13.4, and 33.3 µmol/mL, respectively)

Each ligand solution was added to the glyoxal beads, and the slurries were gently agitated at RT for 4 h. Beads were drained using a vacuum pump aspirator, and pooled filtrates were retained for ligand loading level (LL) analysis. The beads were washed with coupling buffer (5 × PBV) followed by PBS (5 × PBV), and the washed filtrates were collected for LL analysis. Each experiment was performed in six replicates (n = 6).

Following the coupling, the beads were stabilized using sodium borohydride (NaBH₄). For every 1 mL of beads, 10 mg (264.3 µmol) of NaBH₄ in coupling buffer was added and incubated for 30 min at RT in a fume hood. The beads were washed with 25 mM phosphate buffer (pH 7.0; 5 × PBV), followed by deionized water (10 × PBV), and finally stored in PBS (pH 7.4) at 4 °C until use. Unmodified control beads were prepared identically, excluding the ligand coupling step (n = 6).

**S2.2. Scanning electron microscopy (SEM)**

Both unmodified and functionalized beads were freeze-dried using an Alpha 1-4 LSCbasic freeze dryer (Martin Christ GmbH, Germany). beads were snap-frozen in liquid nitrogen for 5 min and then freeze-dried overnight at − 50 °C under 0.1 mbar vacuum. freeze-dried samples were stored at 4 °C in sealed containers. Prior to imaging, the beads were mounted on aluminum stubs with carbon tape and sputter-coated with a ~10 nm platinum layer. Imaging was performed with a Zeiss EVO LS15 scanning electron microscope (Oxford Instruments X-MaxN, Hitachi) at 15 kV.

**S2.3. Fourier-transform infrared spectroscopy (FTIR)**

Freeze-dried, unmodified, and functionalized beads (prepared for SEM) were analyzed using a JASCO FT/IR 4200 spectrometer (JASCO Co., Ltd., Tokyo, Japan). Spectra were collected using the Spectra Manager II software, averaging 20 scans at 4 cm⁻¹ resolution across the 600–4000 cm⁻¹ range.

**S2.4. Leaching and stability studies**

To assess ligand leaching, 1 mL of Hp-, HSA-, and DFO-functionalized beads was incubated in 2 mL of PBS (pH 7.4) at 4 °C or –20 °C for 1, 7, 14, 30, and 60 days. At each time point, the supernatants were recovered via pipetting or centrifugation and analyzed using Pierce 660 nm and BCA protein assays (for Hp and HSA) and 430 nm assay (for DFO). All assays were performed according to the manufacturer’s instructions.

To assess functional stability, Hp-, HSA-, and DFO-functionalized beads were tested at each time point using the QuantiChrom Hb, heme, and iron assays, respectively, as described by the manufacturer (n = 4).

**S2.5. Hemocompatibility studies**

Whole blood from three consenting healthy donors was collected in 0.109 M sodium citrate vacutainers and processed within 1 h. Hp-, HSA-, and DFO-functionalized beads were mixed in a 90:5:5 ratio and packed into 1 mL filter columns.

Citrated whole blood (2 mL) was added to each column containing either the functionalized or unmodified beads and incubated for 30 min at RT. Blood was allowed to pass through the beads under gravity, and filtrates were collected in 15 mL polypropylene conical tubes (Sarstedt, 120 × 17 mm). Samples were held at 4–8 °C for 15 min, then centrifuged at 2000 g for 10 min to isolate plasma. Plasma was aliquoted (1 mL) into 2 mL polypropylene microcentrifuge tubes and stored at - 20 °C (n = 3 per donor).

Human D-dimer and thrombin-antithrombin (TAT) complex ELISA kits were used to assess biocompatibility. Prior to analysis, plasma samples were thawed at 37 °C for 2 min, and the D-dimer concentrations and TAT complexes measured in the plasma samples following manufacturer’s instructions. Absorbance at 450 nm was measured using a Clariostar Plus microplate reader. Measuring ranges were 4.69–300 ng/mL for D-dimer and 1–10 ng/mL for TAT complexes.

**S2.6. Hb binding using the Harboe technique**

Free Hb concentration was quantified using the Harboe spectrophotometric method **[1]**. The samples were diluted 1:10 in 0.1% Na₂CO₃ and added to 96-well plates. Absorbance was measured at 415 nm (oxyhaemoglobin peak), 380 nm, and 450 nm to account for protein and lipid interference, respectively. Measurements were performed using a Clariostar Plus microplate reader and fHb concentrations were calculated using the following equation:

fHb (mg/dL) = 83.6 (2 × A415 nm − A380 nm − A450 nm)

**S2.8. Packed-bed column analysis**

To provide a quantitative framework for interpreting the scale-up experiment, column performance was described using standard packed-bed parameters **[2]**. The empty-bed contact time (EBCT), representing the approximate residence time of fluid within the packed bed, was calculated as:

**EBCT = Vbed / Q**

where **Vbed** is the packed-bed volume and **Q** is the volumetric flow rate.

In this study, 200 mL of hemolyzed whole blood was processed over 30 min through a 250 mL packed bed of functionalized beads under gravity-fed conditions. This corresponded to an approximate flow rate of 6.7 mL/min and an estimated EBCT of approximately 35-40 min. Although time-resolved outlet profiles were not obtained, the column was interpreted as a packed-bed adsorption system operating under gravity-fed, batch-equivalent conditions, where adsorption performance is governed largely by contact time between the fluid phase and immobilized ligands. Binding capacity was expressed as mass of analyte removed per unit bed volume, enabling comparison between small-scale and scaled-up systems.

**S3. Results**

**S3.1. Leaching and stability studies**

**Leaching profile**

Affinity ligand leaching from Hp-, HSA-, and DFO-functionalized glyoxal agarose beads was assessed over 1–60 days of storage in PBS at either 4 °C or − 20 °C (**Table S1**). No significant differences in ligand release were observed across time points or between storage conditions, confirming that covalent immobilization was stable at both storage temperatures.

**a)**

| Storage time  (days) | Initial Hp quantity (mg) | Immobilized Hp (mg/mL) | %Hp Leaching | | Concentration of Hp leaching (mg/mL) | |
| --- | --- | --- | --- | --- | --- | --- |
|  |  |  | **4 °C** | **- 20 °C** | **4 °C** | **- 20 °C** |
| 1 | 58 | 32.7 ± 1.5 | 0.11 ± 0.01 | 0.14 ± 0.04 | 0.12 ± 0.01 | 0.14 ± 0.04 |
| 7 | 58 | 32.7 ± 1.5 | 0.05 ± 0.00 | 0.14 ± 0.01 | 0.05 ± 0.00 | 0.15 ± 0.01 |
| 14 | 58 | 32.7 ± 1.5 | 0.08 ± 0.04 | 0.08 ± 0.06 | 0.08 ± 0.04 | 0.09 ± 0.07 |
| 30 | 58 | 32.7 ± 1.5 | 0.28 ± 0.14 | 0.11 ± 0.01 | 0.29 ± 0.15 | 0.11 ± 0.01 |
| 60 | 58 | 32.7 ± 1.5 | 0.53 ± 0.31 | 0.48 ± 0.22 | 0.54 ± 0.32 | 0.49 ± 0.23 |

**b)**

| Storage time  (days) | Initial Hp quantity (mg) | Immobilized Hp (mg/mL) | %Hp Leaching | | Concentration of Hp leaching (mg/mL) | |
| --- | --- | --- | --- | --- | --- | --- |
|  |  |  | **4 °C** | **- 20 °C** | **4 °C** | **- 20 °C** |
| 1 | 58 | 32.7 ± 1.5 | 0.04 ± 0.00 | 0.03 ± 0.00 | 0.05 ± 0.00 | 0.04 ± 0.00 |
| 7 | 58 | 32.7 ± 1.5 | 0.04 ± 0.00 | 0.02 ± 0.00 | 0.04 ± 0.00 | 0.02 ± 0.00 |
| 14 | 58 | 32.7 ± 1.5 | 0.05 ± 0.02 | 0.05 ± 0.03 | 0.05 ± 0.02 | 0.05 ± 0.03 |
| 30 | 58 | 32.7 ± 1.5 | 0.08 ± 0.02 | 0.07 ± 0.00 | 0.08 ± 0.02 | 0.07 ± 0.00 |
| 60 | 58 | 32.7 ± 1.5 | 0.13 ± 0.01 | 0.10 ± 0.04 | 0.13 ± 0.01 | 0.10 ± 0.04 |

**c)**

| Storage time  (days) | Initial HSA quantity (mg) | Immobilized HSA (mg/mL) | %HSA Leaching | | Concentration of HSA leaching (mg/mL) | |
| --- | --- | --- | --- | --- | --- | --- |
|  |  |  | **4 °C** | **- 20 °C** | **4 °C** | **- 20 °C** |
| 1 | 65 | 51.4 ± 4.7 | 0.12 ± 0.01 | 0.07 ± 0.01 | 0.15 ± 0.01 | 0.09 ± 0.01 |
| 7 | 65 | 51.4 ± 4.7 | 0.06 ± 0.00 | 0.12 ± 0.01 | 0.08 ± 0.01 | 0.15 ± 0.01 |
| 14 | 65 | 51.4 ± 4.7 | 0.03 ± 0.01 | 0.07 ± 0.05 | 0.04 ± 0.02 | 0.07 ± 0.05 |
| 30 | 65 | 51.4 ± 4.7 | 0.23 ± 0.08 | 0.16 ± 0.02 | 0.29 ± 0.10 | 0.20 ± 0.02 |
| 60 | 65 | 51.4 ± 4.7 | 0.19 ± 0.05 | 0.13 ± 0.07 | 0.23 ± 0.07 | 0.16 ± 0.08 |

**d)**

| Storage time  (days) | Initial HSA quantity (mg) | Immobilized HSA (mg/mL) | %HSA Leaching | | Concentration of HSA leaching (mg/mL) | |
| --- | --- | --- | --- | --- | --- | --- |
|  |  |  | **4 °C** | **- 20 °C** | **4 °C** | **- 20 °C** |
| 1 | 65 | 51.4 ± 4.7 | 0.05 ± 0.00 | 0.08 ± 0.00 | 0.06 ± 0.00 | 0.10 ± 0.01 |
| 7 | 65 | 51.4 ± 4.7 | 0.11 ± 0.00 | 0.01 ± 0.00 | 0.14 ± 0.00 | 0.01 ± 0.00 |
| 14 | 65 | 51.4 ± 4.7 | 0.04 ± 0.01 | 0.02 ± 0.01 | 0.04 ± 0.01 | 0.02 ± 0.01 |
| 30 | 65 | 51.4 ± 4.7 | 0.22 ± 0.03 | 0.08 ± 0.00 | 0.27 ± 0.04 | 0.10 ± 0.00 |
| 60 | 65 | 51.4 ± 4.7 | 0.14 ± 0.02 | 0.11 ± 0.01 | 0.17 ± 0.03 | 0.14 ± 0.01 |

**e)**

| Storage time  (days) | Initial DFO quantity (mg) | Immobilized DFO (mg/mL) | %DFO Leaching | | Concentration of DFO leaching (mg/mL) | |
| --- | --- | --- | --- | --- | --- | --- |
|  |  |  | **4 °C** | **- 20 °C** | **4 °C** | **- 20 °C** |
| 1 | 35 | 8.9 ± 2.0 | 0.08 ± 0.00 | 0.12 ± 0.01 | 0.05 ± 0.00 | 0.07 ± 0.00 |
| 7 | 35 | 8.9 ± 2.0 | 0.16 ± 0.00 | 0.02 ± 0.00 | 0.09 ± 0.00 | 0.01 ± 0.00 |
| 14 | 35 | 8.9 ± 2.0 | 0.12 ± 0.04 | 0.09 ± 0.00 | 0.07 ± 0.02 | 0.05 ± 0.00 |
| 30 | 35 | 8.9 ± 2.0 | 0.30 ± 0.05 | 0.14 ± 0.00 | 0.17 ± 0.03 | 0.08 ± 0.00 |
| 60 | 35 | 8.9 ± 2.0 | 0.25 ± 0.01 | 0.20 ± 0.03 | 0.15 ± 0.00 | 0.12 ± 0.02 |

**f)**

| Storage time  (days) | Initial DFO quantity (mg) | Immobilized DFO (mg/mL) | %DFO Leaching | | Concentration of DFO leaching (mg/mL) | |
| --- | --- | --- | --- | --- | --- | --- |
|  |  |  | **4 °C** | **- 20 °C** | **4 °C** | **- 20 °C** |
| 1 | 35 | 8.9 ± 2.0 | 0.23 ± 0.02 | 0.19 ± 0.04 | 0.13 ± 0.01 | 0.11 ± 0.02 |
| 7 | 35 | 8.9 ± 2.0 | 0.11 ± 0.00 | 0.25 ± 0.02 | 0.06 ± 0.00 | 0.15 ± 0.01 |
| 14 | 35 | 8.9 ± 2.0 | 0.16 ± 0.05 | 0.20 ± 0.01 | 0.09 ± 0.03 | 0.12 ± 0.00 |
| 30 | 35 | 8.9 ± 2.0 | 0.48 ± 0.15 | 0.26 ± 0.02 | 0.29 ± 0.09 | 0.15 ± 0.01 |
| 60 | 35 | 8.9 ± 2.0 | 0.65 ± 0.30 | 0.54 ± 0.36 | 0.39 ± 0.18 | 0.32 ± 0.21 |

**Table S1.** Affinity ligand leaching from Hp-, HSA-, and DFO-functionalized glyoxal agarose beads over 60 days under refrigerated (4 °C) and frozen (- 20 °C) storage. Hp leaching was measured using the Pierce 660 nm protein assay **(a)** and BCA assay **(b)**; HSA leaching using the Pierce 660 nm protein assay **(c)** and BCA assay **(d)**; and DFO leaching using the OPA assay **(e)** and spectrophotometric assay at 430 nm **(f)**. No significant differences were observed between refrigerated and frozen samples at any time point.

**Stability profile**

The stability of affinity ligand-functionalized beads was evaluated by monitoring target analyte binding after storage at 4 °C and -20 °C for up to 60 days (**Figure S1**). Hp-functionalized beads showed largely stable Hb binding under both storage conditions. At 4 °C, Hb binding decreased modestly from 11.2 ± 0.7 mg/mL at day 1 to 9.7 ± 0.7 mg/mL at day 30 and 9.3 ± 0.2 mg/mL at day 60, while beads stored at - 20 °C maintained comparable binding across all time points (**Figure S1a**). The HSA-functionalized beads retained high heme-binding capacity after storage at both temperatures. binding values remained within a narrow range over time, with day 60 values of 3296.5 ± 13.3 µg/mL for samples stored at 4 °C and 3387.2 ± 20.8 µg/mL for samples stored at −20 °C, indicating minimal impact of storage conditions on heme-binding performance (**Figure S1b**). Similarly, DFO-functionalized beads exhibited generally stable iron-binding capacity over a 60-day period. Iron binding decreased modestly from initial values of 253.0 ± 21.7 µg/mL (4 °C) and 257.6 ± 19.5 µg/mL (-20 °C) to 218.0 ± 2.6 µg/mL and 227.5 ± 2.2 µg/mL, respectively, by day 60 (**Figure S1c**). Overall, all affinity ligand-functionalized beads preserved their functional binding capacity for at least 60 days under refrigerated and frozen storage conditions, demonstrating good storage stability.

**a)**

**b)**

**c)**

**Figure S1.** Stability of Hp-, HSA-, and DFO-functionalized glyoxal agarose beads over 60 days under refrigerated (4 °C) and frozen (-20 °C) storage, assessed by binding to their respective target analytes. **(a)** Hb binding by Hp-functionalized beads measured using the Quantichrom Hb assay. **(b)** Heme binding by HSA-functionalized beads measured using the Quantichrom heme assay. **(c)** Iron binding by DFO-functionalized beads measured using the Quantichrom iron assay. Data are shown as individual data points (n = 4). Where indicated, statistical analysis was performed using two-way ANOVA to assess the effects of storage condition and time; significance levels are denoted as **P* ≤ 0.05.

**S3.2. Hemocompatibility studies**

The hemocompatibility of the functionalized beads was assessed by quantifying the thrombin-antithrombin (TAT) complex and D-dimer levels in the plasma after 30 min of incubation with functionalized or control unmodified beads (**Figure S2**). The TAT levels remained below the normal reference threshold (<3.0 ng/mL) in all samples (**Figure S2a**), with no significant difference between the groups. Similarly, D-dimer levels remained below 500 ng/mL for both functionalized and control beads (**Figure S2b**), with no significant differences. These findings confirmed that the functionalized beads were hemocompatible and did not induce thrombotic or fibrinolytic activation.

**a)	b)**

**Figure S2.** Concentrations of **(a)** TAT complex and **(b)** D-dimer in plasma from three healthy donors following 30 min incubation with Hp-, HSA-, and DFO-functionalized glyoxal agarose beads, and unmodified control beads. Normal reference ranges: TAT complex <3.0 ng/mL; D-dimer <500 ng/mL. No significant differences were observed between functionalized and control beads for either parameter.

**S3.3. Hemoglobin binding using the Harboe method**

**In human plasma**

Hb binding by Hp-functionalized glyoxal agarose beads was further assessed in plasma using the Harboe method (**Figure S3**). Hb binding increased with increasing Hb concentration (250–5000 µg/mL). At lower concentrations (250 and 500 µg/mL), the binding to the functionalized beads remained limited. At higher concentrations, binding increased markedly, reaching 2.4 ± 0.1 mg/mL (37.2 ± 1.7% binding efficiency) at 1000 µg/mL and 8.6 ± 0.4 mg/mL (51.3 ± 2.3%) at 5000 µg/mL. One-way ANOVA performed across functionalized bead groups confirmed a significant concentration-dependent increase in Hb binding (*P* ≤ 0.0001). These results are consistent with those obtained using the Quantichrom Hb assay.

**Figure S3.** Hb binding by Hp-functionalized glyoxal agarose beads from human plasma spiked with Hb (250– 5000 µg/mL), measured using the Harboe method. Data are shown as individual data points (n = 4). Statistical analysis was performed between functionalized bead groups only using one-way ANOVA; *****P* ≤ 0.0001.

**In hemolyzed whole blood**

The Harboe method confirmed superior Hb binding in Hp-functionalized beads compared with that in the controls (**Figure S4**). At 1000 µg/mL, binding was 2.2 ± 0.6 mg/mL (32.2 ± 9.7%). At 5000 µg/mL, binding reached 8.6 ± 0.5 mg/mL (66.9 ± 4.4%) versus 0.6 ± 0.1 mg/mL for controls (*P* ≤ 0.0001). These results were consistent with those obtained using the Quantichrom method, reinforcing the high Hb-binding performance of the beads in whole blood.

The binding of Hb to Hp-functionalized glyoxal agarose beads in hemolyzed whole blood was evaluated using the Harboe method (**Figure S4**). Hb binding remained low at lower Hb concentrations (250 and 500 µg/mL) and increased at higher concentrations, reaching 2.2 ± 0.6 mg/mL at 1000 µg/mL and 8.6 ± 0.5 mg/mL at 5000 µg/mL. One-way ANOVA across functionalized bead groups confirmed a significant concentration-dependent increase in Hb binding (*P* ≤ 0.0001). Binding by the unmodified beads was minimal across the tested concentration range. These findings are consistent with those of the Quantichrom Hb assay and confirm that Hp-functionalized beads retain their effective Hb-binding capacity in hemolyzed whole blood.

**Figure S4.** Hb binding by Hp-functionalized glyoxal agarose beads from hemolyzed whole blood (250 - 5000 µg/mL), measured using the Harboe method. Data are shown as individual data points (n = 4). Statistical analysis was performed between Hp-functionalized bead groups only using one-way ANOVA; ***P* ≤ 0.01, *****P* ≤ 0.0001.

**In column scale-up study**

In the scaled-up column study, Hb binding, measured using the Harboe method, confirmed the Quantichrom results (**Figure S5)**. Functionalized beads achieved significantly higher binding (10.8 ± 0.4 mg/mL) compared to unmodified beads (2.4 ± 0.3 mg/mL, *P* ≤ 0.0001), demonstrating consistent performance across analytical methods.

**Figure S5.** Hb binding efficiency of Hp-functionalized glyoxal agarose beads in the scale-up study measured by the Harboe method. Functionalized beads (250 mL, 90:5:5 ratio of Hp:HSA:DFO) were incubated with 200 mL hemolyzed whole blood. Data are mean ± SD (n = 4). Statistical significance: *****P* ≤ 0.0001; Student’s *t*-test.

**S4. References**

1. Chung HJ, Chung JW, Yi J, *et al.* Automation of Harboe method for the measurement of plasma free hemoglobin. J Clin Lab Anal. 2020;34(6):e23242.
2. Crittenden JC, Trussell RR, Hand DW, Howe KJ, Tchobanoglous G. MWH's Water Treatment: Principles and Design. 3rd ed. Hoboken: John Wiley & Sons; 2012.
3. Thomas HC. Heterogeneous ion exchange in a flowing system. J Am Chem Soc. 1944;66(10):1664–1666.
4. Hutchins RA. New method simplifies design of activated-carbon systems. Chem Eng. 1973;80(19):133–138.
